# Detailed Physicochemical Analysis of Hyaluronic Acid and Transferrin Self-Assembly To Produce Drug Carrier Colloids

**DOI:** 10.1021/acsomega.5c08615

**Published:** 2025-11-11

**Authors:** László Seres, Norbert Varga, Bianka Torma, Ádám Juhász, Edit Csapó

**Affiliations:** † Interdisciplinary Excellence Center, Department of Physical Chemistry and Materials Science, 37442 University of Szeged, Rerrich B. sqr. 1, Szeged H-6720, Hungary; ‡ MTA-SZTE Lendület “Momentum” Noble Metal Nanostructures Research Group, University of Szeged, Rerrich B. sqr. 1, Szeged H-6720, Hungary

## Abstract

Polyelectrolyte complexes (PECs) are polymeric nanostructures created by the self-assembly of oppositely charged macromolecules. PEC-based novel biomaterials are currently being researched as controlled drug delivery vehicles due to their unique combination of beneficial properties. In this work, the formation of a new colloid system composed of human apo-transferrin (Tr) protein and high-molecular-weight hyaluronic acid (HyA) polysaccharide was studied from numerous perspectives because, to the best of our knowledge, no information is available regarding this PEC-based Tr-HyA formula. For detailed characterization, the experimental results of several physicochemical and colloid chemistry techniques, such as light scattering, rheology, titration microcalorimetry, and electron microscopy, were analyzed. It was emphasized that the pH and mass ratio of the macromolecules greatly influence the self-assembly process. Particles with enhanced stability were prepared at a Tr/HyA = 2:1 mass ratio. The applicability of these colloid particles with a diameter of 240–260 nm as drug delivery vehicles was evaluated by encapsulating several practically water-insoluble molecules to increase their solubility in water. We highlighted that the nanoformulation revealed ca. 3 times better solubility and enhanced release for vitamins D3 and K1 compared to the unformulated ones.

## Graphical abstract

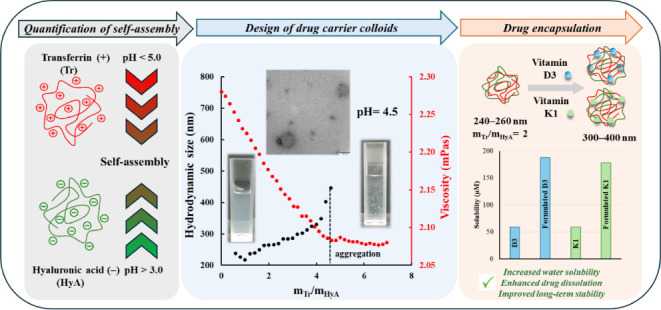

## Introduction

Nano- or colloidal particles can be used as drug carrier capsules to provide fewer side effects, better efficiency, more suitable dosing, better distribution in the body, or targeting to a specific point. Among liposomes,^1^ micelles,^2^ inorganic particles,^3^ and emulsions,^4^ the (bio)­macromolecular formulations have great importance in creating colloidal capsules. The use of polysaccharides (like chitosan, alginate, or hyaluronic acid) as building units of drug delivery systems is especially favored due to their high biocompatibility.^5^ The hyaluronic acid (HyA) consists of repeating disaccharide groups of *D*-glucuronic acid and *N*-acetyl-*D*-glucosamine.^6^ The functional groups of the monomers give the polyelectrolyte excellent water solubility and thus a strong hydrophilic character. This is the main reason why, in most cases, HyA alone is not efficient enough to create a drug delivery system, so either the chemical modification^7^ or the interaction with different materials is needed. The chemical modification can be done through the carboxylic group of the polysaccharide^8^ with ester or amide formation, as well as on the −NHCOCH_3_ group^9^ with deacetylation or on the −OH group^10,11^ with ester formation. However, these reactions often need different coupling agents and organic solvents, of which any remaining in the final product can significantly decrease the excellent biocompatibility of HyA. Since HyA has a negative charge in a wide pH range (pH > p*K*_a, monomer_ ≈ 3.0),^12^ the aforementioned problem can simply be overcome by the cross-linking of the polymer with an oppositely charged, biocompatible material. Several studies have shown that the electrostatic forces between HyA and positively charged (macro)­molecules can result in the formation of colloidal particles, also known as polyelectrolyte complexes (PEC), which are sufficient for drug delivery.^13^ In addition to their numerous advantageous properties, such as biocompatibility, biodegradability, and mild and cost-effective preparation conditions, the pH range where they lose their opposite charge and maintain their stability presents challenges. However, the stability of the charge-compensated systems in physiological conditions is low due to the high ionic strength in biological fluids; this feature can be greatly improved by using gastroprotective capsules^14^ or gel matrix^15^ as an additional protection layer. The most well-known positively charged materials used with HyA are zein,^16^ chitosan,^17,18^ or polyarginine.^19^ In the case of proteins, the charge ratios are not as evident as in the case of HyA. Since they are made of amino acids whose side chains are capable of ionization, they can have an overall positive or negative charge as well, depending on the pH of the medium. The charge-neutral pH value is also called the isoelectric point (pI), under which the protein has a grossly positive charge. According to these, positively charged proteins, such as lysozyme (Lys, pI = 10.7),^20,21^ bovine,^22^ or human serum albumin (BSA and HSA pI = 5.1 and 4.7)^23^ can be used to create PEC complexes. The common feature of these proteins is that at pH values under the isoelectric points, they have a positive charge; therefore, spontaneous complex formation with HyA is achievable. The same phenomenon is valid for human serum transferrin (Tr), as well. Tr is a glycoprotein and has a molecular weight of ∼80 kDa. The iron-free form of this macromolecule is also called apo-transferrin, which has an isoelectric point of ∼ 5.6.^24^ In tumor cells, the Tr receptor is overexpressed, which makes cancer cells more sensitive to Tr itself, and the Tr uptake of these cells is greater than that of normal ones. This phenomenon can increase the importance of planning, synthesizing, and characterizing Tr-modified drug delivery systems in the fight against cancer.^25^ In many cases, covalent bonding of the protein to a macromolecule^26–28^ or colloidal particles, e.g., liposomes^29^ is carried out, resulting in the formation of drug delivery systems; however, electrostatic binding of Tr to macromolecules is rarely reported.

Our previous experience with the topic^30^ revealed that in the case of serum proteins, complex formation with HyA is possible. To the best of our knowledge, there has not been any scientific report written about the interpretation of the self-assembly of Tr and high-molecular-weight HyA to create stable colloidal particles. Although there are plenty of publications in the field of PECs, the detailed mapping of a system, which has never been done before, is really important, since the main features of the created system cannot be simulated or estimated only by interpreting data from existing publications. This is why the primary motivation of our work was to quantify this self-assembly process using several modern physicochemical and colloid chemistry techniques. The encapsulation of water-insoluble molecules (artesunate, quercetin, curcumin, Vitamin K1, and Vitamin D3) as model hydrophobic drugs was also carried out in these newly designed complex Tr-HyA particles to prove their solubilization capacity. The results were compared to one of our previous similar systems composed of human serum albumin (HSA) and HyA.^23^ Furthermore, the drug dissolution processes were also analyzed.

## Experimental Section

### Materials

Hyaluronic acid sodium salt (HyA, (C_14_H_21_O_11_N)_*n*_, *M*_w_ = 1.5–1.8 × 10^3^ kDa, ≤1% protein), human apo-Transferrin (Tr, *M*_w_ = 80 000 Da, 98%, agarose gel electrophoresis, suitable for cell culture), Vitamin D3 (D3, cholecalciferol, C_27_H_44_O, *M*_w_ = 384.64 g/mol, ≥98% (HPLC)), Vitamin K1 (K1, phytonadione, C_31_H_46_O_2_, *M*_w_ = 450.70 g/mol, Pharmaceutical Secondary Standard), curcumin (Cur, C_21_H_20_O_6_, *M*_w_ = 368.38 g/mol, ≥65%), quercetin hydrate (Que, C_15_H_10_O_7_ × H_2_O, *M*_w_ = 302.24 g/mol, ≥95%), artesunate (Art, C_19_H_28_O_8_, *M*_w_ = 384.42, Pharmaceutical Secondary Standard), hydrochloric acid (HCl, 37%) and Triton X-100 (TX-100, C_14_H_22_O­(C_2_H_4_O)_*n*_, laboratory grade) were purchased from Sigma-Aldrich, Hungary. Sodium acetate (CH_3_COONa × 3 H_2_O, ≥99%), acetic acid (CH_3_COOH, ≥96%), sodium chloride (NaCl, ≥99%), sodium phosphate dibasic dodecahydrate (Na_2_HPO_4_ × 12 H_2_O; ≥99%), sodium phosphate monobasic dihydrate (NaH_2_PO_4_ × 2 H_2_O; ≥99%), sodium hydroxide (NaOH; 99.80%) and ethanol (C_2_H_6_O, 99.98%) were obtained from Molar Chemicals Kft., Hungary. Potassium dihydrogen phosphate (KH_2_PO_4_, ≥99.5%) was obtained from Reanal. Highly purified water was obtained by deionization and filtration with a Millipore purification apparatus (18.2 MΩ·cm at 25 °C). All solvents and reagents used for preparation were of analytical grade, and no further purifications were made. The dialysis of the prepared particles was carried out with the help of a semipermeable cellulose dialysis bag (*M*_w_ cutoff = 14 000; Sigma-Aldrich).

### Preparation of the Tr-HyA Particles

First, 1 mg/mL stock solution of HyA was prepared in acetate buffer (pH = 4.5, I = 15 mM). To enhance the dissolution of the polysaccharide, the solution was stirred at 500 rpm for at least 1 h and stored at 4 °C overnight. Before preparation, the HyA stock solution was diluted to a concentration of 0.05 mg/mL (*V* = 5 mL), and a Tr solution of 5 mg/mL in acetate buffer was prepared freshly and stirred for 30 min at 500 rpm to ensure complete dissolution of the protein. The preparation of the particles was carried out by titrating the diluted HyA solution with 100 μL of protein solution at a dosing speed of 10 μL/20 seconds and 500 rpm magnetic stirring, thus obtaining a particle dispersion with *m*_Tr_/*m*_HyA_ = 2 mass ratio. The titrating solution was the concentrated Tr solution, since it has significantly lower viscosity than a HyA solution of the same concentration, making the titrations more precise. After preparation, the dispersion was stirred for 1 h. To demonstrate the success of the small-scale (4–5 times) scale-up, 20 mL of HyA was titrated with 400 μL of Tr solution under the same circumstances, as mentioned above. The volume increment did not have a significant effect on the size of the particles, as shown in Figure S1. If a solid sample was needed, the particles were centrifuged at 15 000 rpm for 1 h and the supernatant was removed. After that, the sample was frozen, lyophilized, and stored at −20 °C. The drug-loaded particles were prepared similarly; however, the drugs were dissolved in ethanol (5, 10, 20 mg/mL). From these stock solutions, 100 μL was added to 300 μL, 6.67 mg/mL Tr solution (*c*_Tr, final_ = 5 mg/mL). After the addition of the drug-containing protein solutions, the dispersions were stirred for at least 1 h. Finally, 20 mL of 0.05 mg/mL HyA solution was titrated with 400 μL of the above-mentioned Tr-drug dispersion at 10 μL/20 s dosing speed and at 500 rpm magnetic stirring. The centrifugation and lyophilization steps were carried out similarly to the empty particles. The representative preparation steps and final solid products are shown in Figure S2 for Vitamin D3.

### Light Scattering and ζ-Potential Measurements

Dynamic light scattering (DLS) and ζ-potential (electrophoretic light scattering) measurements were carried out with a HORIBA SZ-100 NanoParticle Analyzer (HORIBA Jobin Yvon, Longjumeau, France), equipped with a semiconductor laser (λ = 532 nm, 10 mW) as the light source and a photomultiplier detector for quantifying scattered intensity at a 90° scattering angle with a count rate of at least 1000 kCPS for every sample (to obtain more than 1 000 000 counts^31^ within 30 s of accumulation time—Figure S3—during the measurements). With this method, the correlation function did not fluctuate at all by the end of the measurement (stable data points were evaluated), and during every measurement (even at the most turbid ones during the encapsulation), the count rate was at least 1000 kCPS (namely, 1290 ± 91 kCPS for the 20 mg/mL Vitamin K1 and 1737 ± 112 kCPS for the Vitamin D3-containing samples). For the solvent viscosity, the following temperature-dependent eq 1 of water (since a dilute buffer was used as the medium) was applied at 25 ± 0.2 °C (η = 0.898 mPa·s):

1 $$\eta =\left(2.633\times 10^{-8}\right)T^{4}-\left(3.610\times 10^{-5}\right)T^{3}+\left(1.863\times 10^{-2}\right)T^{2}-4.293T+3.736\times 10^{2}$$

For every sample, at least 5 parallel measurements were made. The equilibration time was chosen by the instrument until a stable laser signal was reached (usually 5 s). As a result, hydrodynamic diameter, size distribution, and ζ-potential of the particles were measured. Measurements were carried out at pH levels of 3.6, 4.0, and 4.5, at a a HyA concentration of 0.05 mg/mL. The protein solution (5 mg/mL) was added to the polymer solution in 10 μL portions. The results represent the averages of five measurements. The particle size values were not corrected for the viscosity of the HyA solution at the titration points since the difference was within experimental error (∼5% or less, Figure S4). To ensure that the measurements were of good quality, representative correlation functions of the prepared particles under different conditions are shown in Figure S5. For the same reason, ζ-potential and electrophoretic mobility distribution functions are provided in Figures S6–S8 under different experimental conditions. For the conversion of electrophoretic mobility to ζ-potential, the Smoluchowski model was used (in every case κR > 20).

### Rheology

The apparent viscosity of the Tr-HyA dispersions was determined with an Anton Paar ViscoQC 300 rheometer equipped with a C-DG26 concentric measuring head. The measured curves were determined at 25 ± 0.1 °C and at a shear rate of 250 1/s. Fourteen mL of the diluted HyA solution (0.05 mg/mL) was pipetted into the measurement cell, which was titrated with the Tr solution (5 mg/mL) in 25 μL units/step. With these parameters, 2 parallel measurements were carried out at pH values of 3.6, 4.0, and 4.5.

### Turbidimetry

Turbidimetric titrations were carried out with an LP2000 Hanna Instruments Precision Bench Turbidity Meter LP2000 (Hanna Instruments, Hungary). During the titrations, the protein solution (5 mg/mL) was added to the polymer solution (0.05 mg/mL) in 10 μL portions at three different pH values (pH = 3.6, 4.0, 4.5). The results represent the averages of five measurements.

### Streaming Potential Measurements

When electrically charged species are present in the sample, a Particle Charge Detector (PCD) can supply sufficient information about the charge ratios in the system. To carry out these measurements, a Mütek Particle Charge Detector PCD-04 model (BTG Instruments GmbH, Germany) instrument was used. For the determination of the charge ratios of HyA and the isoelectric point of Tr, streaming potential measurements were carried out. Both macromolecules were dissolved in HCl solution with a pH of 3.0 at *c*_HyA_ = 0.36 mg/mL and *c*_Tr_ = 0.30 mg/mL concentration, respectively, from which 10 mL was titrated with 0.05 M NaOH in 20 μL portions, and the streaming potential and pH of the solution were measured. For the investigation of the interaction between the macromolecules, 10 mL of the 0.05 mg/mL of HyA solution was taken into the sample holder, which was titrated with the Tr solution (5 mg/mL) in 10 μL units/step at three different pH values (pH = 3.6, 4.0, 4.5). The curves were fitted with the modified Boltzmann equation to obtain the inflection points.^18^

### Fourier Transform Infrared Spectroscopy (FT-IR)

FT-IR spectra were registered with a Jasco FT/IR-4700 spectrometer equipped with an ATR Pro One measuring head. Besides the pure solid HyA and Tr, the spectrum of the complex particles created at *m*_Tr_/*m*_HyA_ = 2 mass ratio was also recorded. The sample was freeze-dried before the measurement, which was carried out in the 500–3700 cm^–1^ range with 1 cm^–1^ resolution with the average of 15 spectra. The pH of the solid samples was kept consistent to avoid the spectral changes caused by ionization.

### Differential Scanning Calorimetry (DSC)

DSC curves of the solid samples (HyA, Tr, and lyophilized Tr-HyA (*m*_Tr_/*m*_HyA_ = 2)) were recorded with a Mettler-Toledo 822e calorimeter between 25 and 500 °C using a 5 °C/min heating speed and 50 mL/min N_2_ flow. The results were evaluated with STARe 12.10 software.

### Circular Dichroism Spectroscopy (CD)

CD spectra of the Tr dissolved in pH = 4.5 acetate buffer and the Tr-HyA conjugates in the same medium were recorded with a Jasco J-1100 CD spectrometer at 25 °C in a 1 cm optical path length cuvette. The spectra were acquired at a 100 nm/min scanning speed in the middle UV range (200–300 nm) with a nitrogen flow rate of 3 L/min N_2_ flow. The light source was a high-energy Xe lamp (450 W). The protein solution and the particle dispersion were diluted to 0.02 mg/mL protein concentration, which was determined by a series of protein dilutions (Figure S9). The final spectra are the results of 3 parallel measurements. To quantify the ratio of secondary structures in the protein, the Reed model was used to fit the spectra, which is built into the software of the instrument.

### Isothermal Microcalorimetry (ITC)

The isothermal microcalorimetric titrations were performed in a temperature-controlled room with a MicroCal VP-ITC instrument, which was equipped with a sample cell of 1.4301 mL and a 280 μL syringe. After thoroughly cleaning and rinsing the sample cell, 1.4301 mL of 0.05 mg/mL HyA was loaded into the sample holder and titrated with 280 μL Tr solution (1.00 mg/mL at pH = 3.6, 1.40 mg/mL at pH = 4.0, and 1.875 mg/mL at pH = 4.5) at a dosage speed of 10 μL/5 min after waiting for the temperature to stabilize. The equipment measures the heat that is absorbed or evolved when the Tr solution is dropped into the polysaccharide solution. The initially obtained d*Q*/d*t* chart is converted to an enthalpogram by peaks of the raw data. This integration was automatically made by the Origin Microcal 7.1 software. The measurements were carried out 2 times.

### Dialysis, Conductometry, and High-Resolution Transmission Electron Microscopy Measurements (HR-TEM)

To get acceptable quality TEM images of the particles, dispersions (*m*_Tr_/*m*_HyA_ = 2) were made according to the procedure presented in the Preparation of the Tr-HyA Particles. After preparation, 4 mL of this dispersion was taken into a dialysis bag (*M*_w_ cutoff = 14 000; Sigma-Aldrich) which was immersed in 45 mL of MQ water to eliminate most of the buffer salts. The specific conductance of the MQ water was monitored using a Hanna HI-5321 conductometer with a cell constant of 1.0534 cm^–1^. After 1 h of dialysis, 5 μL of Tr-HyA dispersion (*m*_Tr_/*m*_HyA_ = 2) was dripped onto a copper TEM grid and placed under an infrared lamp to evaporate the solvent. For the TEM measurements, S160 Carbon Film 200 mesh Cu grids were used without preliminary plasma treatment. The TEM pictures were taken with a FEI Tecnai G2 20 X-Twin HR-TEM with an accelerating voltage of 200 kV. In the case of the drug-loaded particles, the TEM pictures were taken with a JEOL Jem-1400Plus electron microscope at accelerating voltage of 120 kV.

### Stability of the Particles in Different Simulated Body Fluids

The stability of the prepared particles was tested in simulated gastric fluid (SGF, pH = 1.2), simulated intestinal fluid (SIF, pH = 6.8), and phosphate-buffered saline (PBS), as well as with the help of dialysis. Namely, 5 mL of the freshly prepared particles was placed into a dialysis bag, which was inserted into 100 mL of the different media, mentioned before. After an hour of dialysis, the samples were taken out of the bag and light scattering studies were carried out (Table S1).

### Determination of Encapsulation Efficiency (EE%) and Drug Loading (DL%)

The determination of EE% and DL% was performed with the help of a JASCO V-770 UV–VIS spectrophotometer. In short, the lyophilized drug-containing Tr-HyA particles were redispersed in ethanol using 10 min of sonication. After this, the samples were stirred for 2 h and centrifuged for 10 min at 10 000 rpm to remove the undissolved materials. The UV–vis spectra of the solutions were recorded in the 200–500 nm range, and the concentration of the drug was determined at the absorption maxima (λ_max, D3_ = 270 nm, λ_max, K1_ = 330 nm, λ_max, Que_ = 374 nm, λ_max, Cur_ = 423 nm) with the help of calibration curves (Figures S10–S13). In the case of Art, the absorption maximum could not be determined within the examined spectrum range, so the calibration was carried out using a differential scanning calorimeter. The DSC curves of different amounts of Art were recorded, and the integral of the peak between 143 and 168 °C was used to create the calibration curve (Figure S14). The EE% and DL% values were determined with the following eqs 2–3.

2 $$\mathrm{EE}\%=\frac{\mathrm{encapsulation}\;\mathrm{mass}\;\mathrm{of}\;\mathrm{drug}}{\mathrm{total}\;\mathrm{mass}\;\mathrm{of}\;\mathrm{drug}}\times 100$$

3 $$\mathrm{DL}\%=\frac{\mathrm{encapsulation}\;\mathrm{mass}\;\mathrm{of}\;\mathrm{drug}}{\mathrm{total}\;\mathrm{mass}\;\mathrm{of}\;\mathrm{nanoparticles}}\times 100$$

### Drug Dissolution Studies

These studies were carried out for K1 and D3, since successful encapsulation was observed for these compounds. In the case of the pure drugs, 0.5 mg of Vitamin K1 and 0.5 mg of Vitamin D3 were dissolved in 40 mL of PBS buffer (pH = 7.4, NaCl (0.9%)) at 37 °C, which also contained 5 mg/mL TX-100 to facilitate the solubility of the drugs. For the formulated K1 and D3, particles with the lowest drug content (5 mg/mL ethanolic drug stock solution) were made according to the procedure presented in Preparation of the Tr-HyA Particles, and these solid samples were dissolved in 40 mL of the buffer. The amount of the particles was chosen to obtain approximately the same drug content in the system as in the case of the pure materials. During the dissolution measurements, 2 mL samples were taken from the systems and replaced with 2 mL of warm PBS buffer containing pure TX-100. The samples were then centrifuged to get rid of possible scattering centers, and the UV–vis spectrum of the samples was recorded at given time intervals for 360 min. The amount of dissolved drug was determined using calibration curves (λ_max, D3_ = 255 nm, λ_max, K1_ = 331 nm) (Figures S15, S16).^32^ Since the calibration solutions contained TX-100 in large amounts, and this surfactant has absorbance peaks between 200–240 and 255–291 nm, the spectra of D3 are noisy in these regimes due to the surfactant. However, the peak at 255 nm was found to be perfect for the creation of the calibration curve, as shown in Figure S15A, B. The vitamins most probably dissolve as free vitamin molecules, which is further confirmed by the similarity between the raw and normalized calibration curve and the UV–vis spectrum of the released vitamin after 20 min (as shown in Figure S17 for K1).

## Results and Discussion

### Determining the Charge of the Macromolecules

During these studies, pH-dependent streaming potential measurements were carried out for both the free HyA and Tr separately (Figure 1A). This technique is relatively less known and used for the quantitative determination of the charge of macromolecules; thus, in this work we further strengthen its applicability.

**Figure 1:**
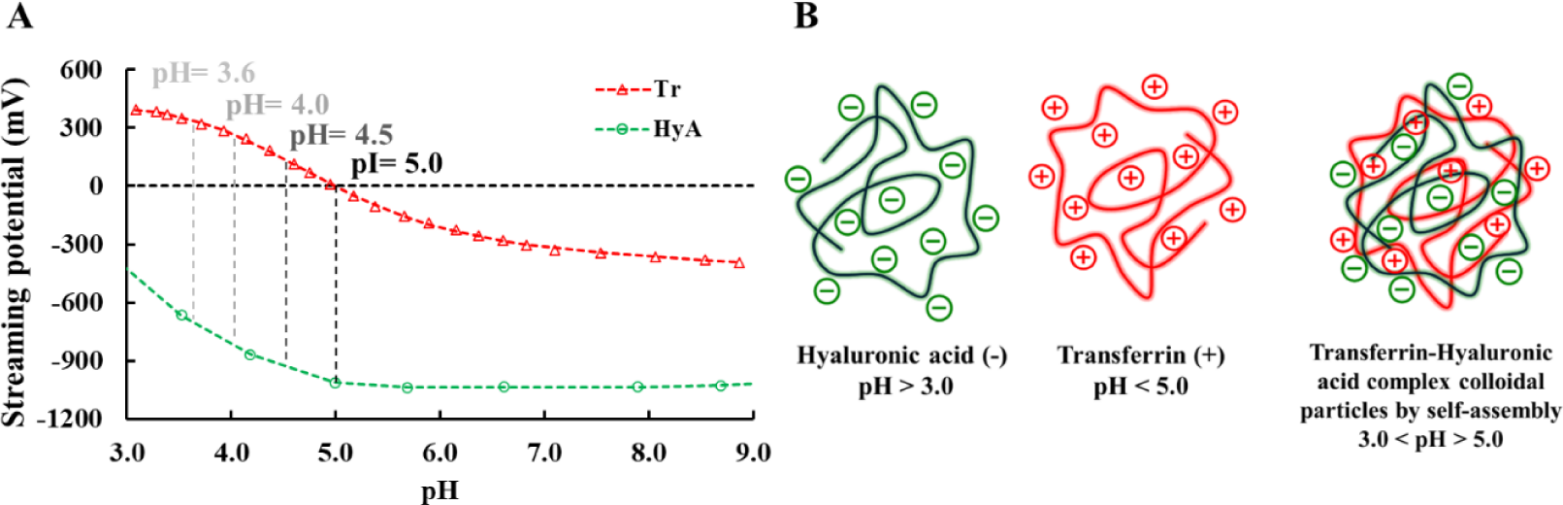
(**A**) Streaming potential measurements for the determination of charges of HyA (○) and Tr (Δ) as a function of pH (*c*_HyA_ = 0.36 mg/mL, *c*_Tr_ = 0.30 mg/mL) and (**B**) schematic representation of the formation of polyelectrolyte complex (PEC) from oppositely charged macromolecules.

In the case of Tr at pH values below 5.00, a positive streaming potential can be measured, which indicates that the protein has a gross positive charge. If the streaming potential curve reaches the 0 point, then Tr has a neutral charge, which means that it is at its isoelectric point (pI). Further titration of the protein solution with NaOH results in the charge reversal of Tr, indicating that more negative charges appear on the macromolecule than positive ones. In contrast, since HyA has a p*K*_a_ at around 3,^12^ its streaming potential curve goes well beyond 0 in all the investigated pH ranges, confirming the statement that HyA has a negative charge due to the deprotonation of its carboxylic groups. These measurements reveal that below the pI of Tr and above the p*K*_a_ of HyA (3.0 < pH > 5.0), electrostatic charge compensation can occur between the macromolecules, which can possibly lead to the formation of PEC, as Figure 1B represents. At different pH values (pH < 2 or pH > 5), the particles can easily disintegrate, which can be beneficial for, e.g., the easier release of encapsulated drugs. To prevent the disintegration of particles in the human body, PEC-based formulations are always placed in gastroprotective capsules^14^ or polymer matrices,^15^ as mentioned earlier, ensuring that the formulation only disintegrates at the target site.

### Physicochemical Analysis of the Self-Assembly of Tr and HyA with Different Measurement Methods

In order to get a comprehensive view of the self-assembly process, the interaction of the two macromolecules was studied by using a number of techniques. In all cases, the HyA solution (*c*_HyA_ = 0.05 mg/mL) was titrated with the Tr solution (*c*_Tr_ = 5 mg/mL) and changes in several characteristics were monitored . First, DLS measurements were performed to examine the size of the forming PECs as a function of the mass ratio of Tr and HyA. We used this form of representation (*m*_Tr_/*m*_HyA_) instead of molar ratios because hyaluronic acid has a molecular mass within such a wide range (*M*_w_ = 1.5–1.8 × 10^3^ kDa) that estimating the moles of the macromolecule based only on an average molecular mass would result in a significant error. Moreover, while the use of charge ratios would also be a rational choice, in our case it is very hard to experimentally quantify the exact number of positive and negative charges present on the protein at a given pH value; thus, we chose mass ratios as the basis for our representations. As shown in Figure 2A, the size of the particles falls within the range of colloidal particles with appropriate polydispersity indices (PDI < 0.3) (Figure S18). It can also be stated that the addition of the protein to the polysaccharide results in an increase in particle size at all pH values (from 226 to 330 nm at pH = 4.5 in the *m*_Tr_/*m*_HyA_ = 1.0–4.0 mass ratio range; see Figure S19B); however, the lower the pH of the medium, the sooner the aggregation of the particles happens (Figure 2A dashed lines).

**Figure 2:**
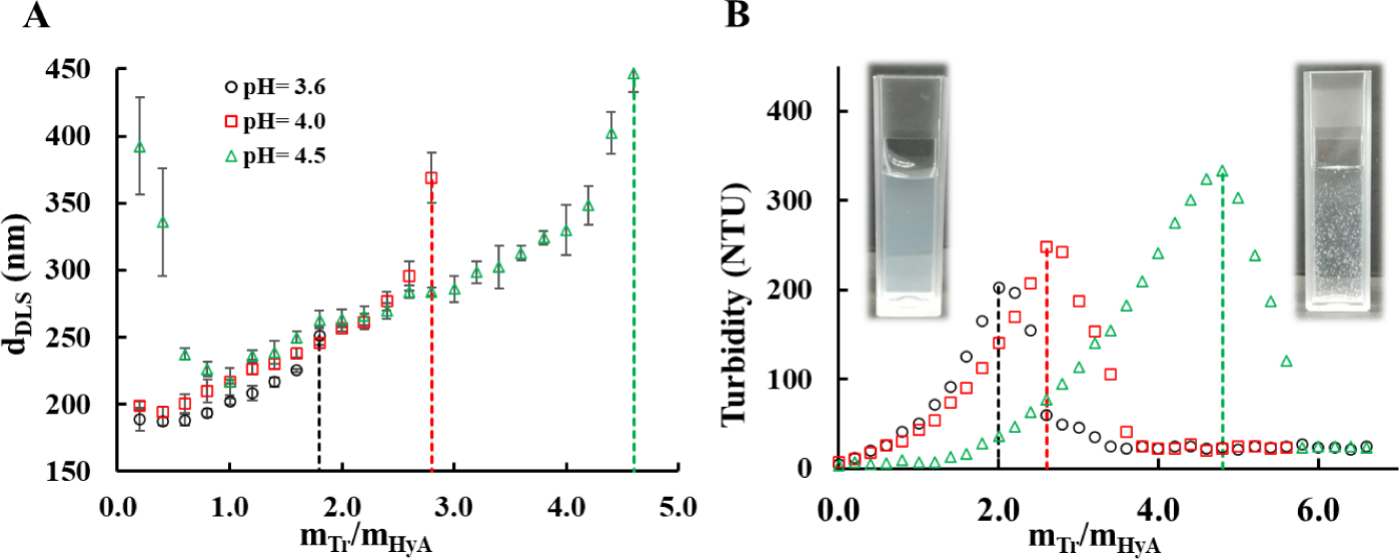
(**A**) The hydrodynamic diameter of the Tr-HyA particles as a function of the mass ratio of the macromolecules at different pH values and (**B**) the turbidimetric curves of the system at different pH values (*c*_HyA_ = 0.05 mg/mL, *c*_Tr titrating_ = 5 mg/mL).

This aligns with the assumption that the higher the pH, the less positive the protein and the more negative the polysaccharide. Furthermore, if we examine the particle size at a certain mass ratio (*m*_Tr_/*m*_HyA_ = 1.2) (Figure S19A), the size of the particles increases to a small extent (from 208 nm at pH = 3.6 to 236 nm at pH = 4.5). The same trend can be observed in the case of turbidimetric studies (Figure 2B). During the titrations, the turbidity of the samples increases due to the formation of more and more colloidal particles, and after reaching a maximum, the turbidity decreases as the particles aggregate and sediment, which reduces the opalescence of the dispersion. The curves also indicate that at higher pH values, more turbid colloidal dispersions can be prepared just before the aggregation (maximum peaks of the curves), which may occur due to the larger particle sizes at the aggregation points.

To avoid the possibility of multiple scattering, a dilution series (no dilution and 2, 4, 8, 16, 32, 64, and 100 times dilution) was prepared for the particles (*m*_Tr_/*m*_HyA_ = 2) to check if the particle size changes. As shown in Figure S20A, the dilution of the system does not have a significant effect on the mean size of the particles; it fluctuates only at around 270 nm. However, in the case of 64 and 100 times dilution, higher particle sizes can be measured (at 100 times dilution, the instrument could not provide any evaluable results), which can be attributed to the worse fitting of the correlation function. Besides these values, we also considered the evaluation of the correlation functions. We can see (Figure S20B) that the correlation functions start to get noisier, and the intercept becomes lower as we dilute the system, which implies that the quality of the measurements worsens with dilution, and the mean sizes obtained from the fitting of the curves become more unreliable. This measurement proved that multiple scattering is not a significant factor for the determination of the size of Tr-HyA particles at *m*_Tr_/*m*_HyA_ = 2. Still, it can be an important parameter for higher *m*_Tr_/*m*_HyA_ ratios; however, at these points the absolute value of the size itself is not an important feature; the values are only needed for the determination of the aggregation points.

Electrophoretic light scattering titrations were performed similarly to the DLS measurements, which revealed that until aggregation occurs, negatively charged particles can be synthesized, as indicated by the negative ζ-potential values at all chosen pH values (Figure 3A).

**Figure 3:**
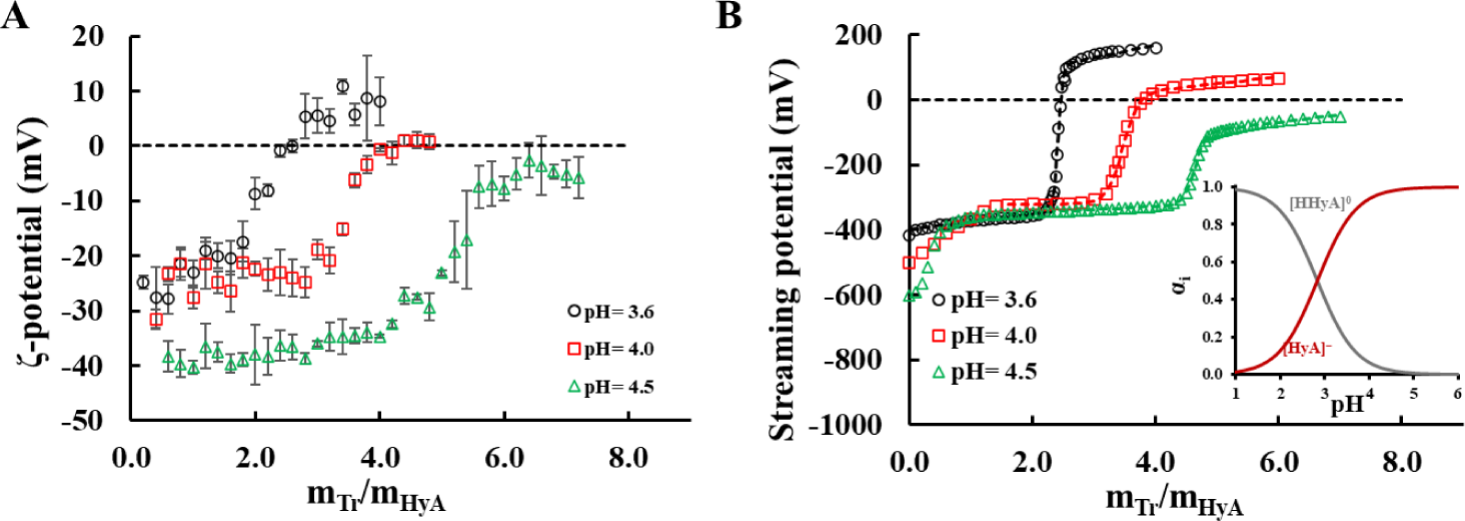
(**A**) ζ-potential and (**B**) streaming potential titration curves of the Tr-HyA system at different pH values (*c*_HyA_ = 0.05 mg/mL, *c*_Tr titrating_ = 5 mg/mL), along with the calculated concentration distribution functions of HyA (inset figure, p*K*_a monomer_ = 3.0 ^12^).

It can also be stated that when aggregation occurs, the ζ-potential of the particles starts rising sharply toward 0, or above it, depending on the pH. This can be explained by the fact that at lower pH values, Tr has more positive charges; thus, after aggregation, it can cause charge reversal in the system. However, at pH = 4.5, the protein has fewer positive charges, so overcharging or charge compensation cannot occur even after aggregation at higher protein excess. Also, we can see in Figure S6 that a single-peak distribution (with a width of around −15 mV at the base of the functions at *m*_Tr_/*m*_HyA_ = 2) of the ζ-potential values was measured for every sample. The only exception was at the *m*_Tr_/*m*_HyA_ = 7 point, which is well after the aggregation of the particles. In this case, one can expect additional peaks to appear at the distribution functions; however, since this regime of the measurements is not important for the creation of stable colloidal particles, we did not use these values further in the publication. The same trend can be observed in the streaming potential curves (Figure 3B), which also highlights the importance of the initial charging state of the polysaccharide. According to the measurements, the higher the pH, the lower the initial streaming potential of HyA is, which is in great agreement with the trends of the deprotonation degree of the polymer, as shown by the inset in Figure 3B. These studies revealed that the most stable particles (with the lowest ζ-potential) can be prepared at pH = 4.5 across the widest mass ratio range. The Tr-HyA system was also characterized from rheological aspects (Figure 4).

**Figure 4:**
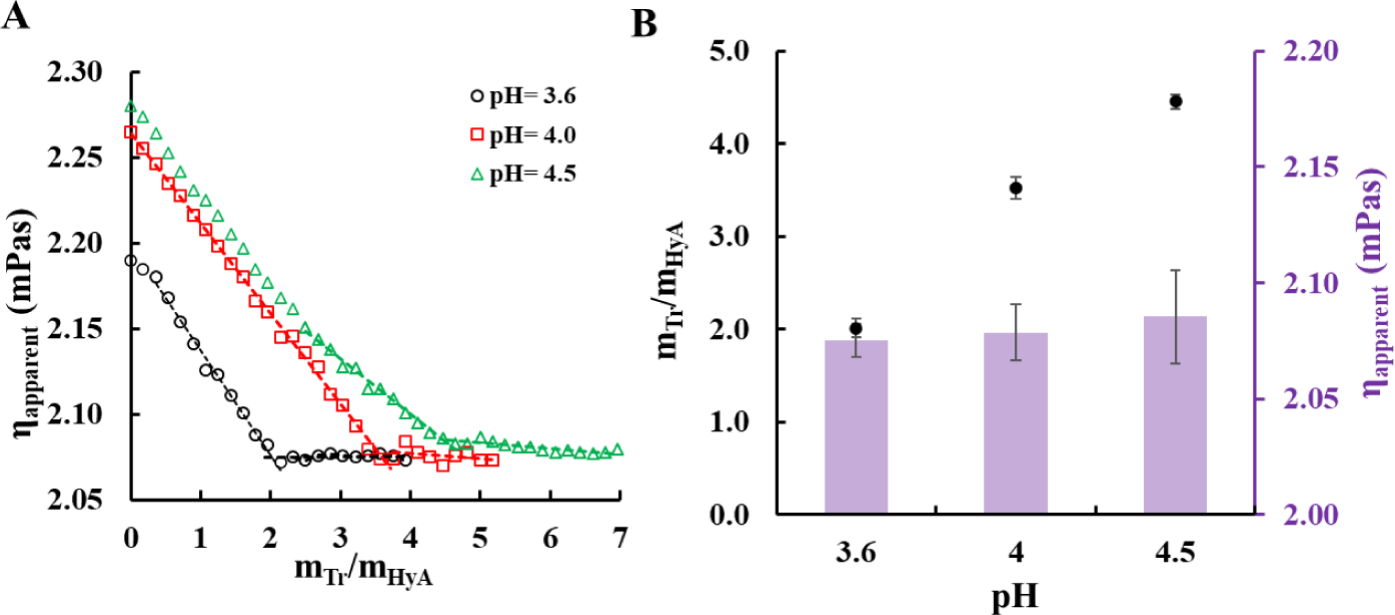
(**A**) Rheological titration curves of the Tr-HyA system at different pH values and (**B**) the values of the Tr-HyA mass ratios (•) and apparent viscosity (columns) at the breaking points as a function of pH (*c*_HyA_ = 0.05 mg/mL, *c*_Tr titrating_ = 5 mg/mL).

According to Figure 4A, the initial stage of the viscosity curves, one can see that an increase in the pH results in a more viscous polymer solution; however, the extent of the change is relatively small. Furthermore, the addition of Tr to the HyA causes a decrease in the viscosity of the dispersion at every pH value, which can be explained by the breaking of the coherent structure of HyA and the formation of individual colloidal particles. The decrease in the viscosity of the medium continues until the aggregation of the particles, which can be seen as a breaking point in the viscosity plot. The values of the mass ratio of the polymers and the viscosity of the medium at these distinct points can be seen in Figure 4B. The shift of these breaking points toward higher mass ratios with an increase in pH is in good agreement with the results of the measurement techniques mentioned before; however, the viscosity of the medium at the aggregation points is the same in every case.

To make our investigation more complete, isothermal microcalorimetric (ITC) titrations were performed as well (Figure S21) During these measurements, the HyA solution (*c* = 0.05 mg/mL) was titrated with Tr. The difference in the initial titrating protein concentrations was necessary due to the fixed volume parameters of the instrument; otherwise, less detailed measurements could have been made. First, the raw calorimetric data were recorded during the titrations of HyA with Tr at different pH values (Figure S21A,C,E). The integration of the individual peaks resulted in obtaining the enthalpy change in the system in one step of Tr addition, for which the enthalpograms at different pH values were calculated (Figure S21B,D,F). Since these measurements provided small d*Q*/d*t* signals, only the main trends of these measurements are discussed. From the thermograms, we concluded that at these concentrations, no significant interactions are present between the macromolecules; however, other methods show that this is not true. After the individual integration of the peaks, small kinks in every enthalpogram can be seen, which consecutively appear at the mass ratios attributed to the aggregation of the particles by several other techniques. Although these trends match well with the results obtained earlier and can give an initial estimation of the aggregation of the particles, due to the small peaks in the thermograms, these results need to be handled carefully. This is why calculating any kind of thermodynamic functions or binding constants from these data or stating far-reaching conclusions based on them would not be appropriate and would result in significant errors; therefore, none of these calculations were carried out.

To sum up the results of all of the above-mentioned measurements, Table 1 shows the characteristic points of the different techniques related to the aggregation of the particles. It can be seen that since each method measures a different feature of the system, the values presented in Table 1 may deviate from one another ; however, the same trend is evident across all of them: the lower the pH of the medium, the sooner the charge compensation of the macromolecules happens, which can be explained well by the different charge states of the polymers mentioned earlier. As a result of the measurements, since the most stable particles (ζ-potential ≈ −38 mV) having ∼263 nm of hydrodynamic diameter can be created at pH = 4.5, this pH value was selected for our further investigations.

**Table 1:** Characteristic Mass Ratio Points (*m*_Tr_/*m*_HyA_) Determined for the Tr-HyA System from the Results of Different Measurement Techniques at Various pH Values

	pH = 3.6	pH = 4.0	pH = 4.5
DLS	1.80	2.80	4.60
Turbidimetry	2.00	2.60	4.80
Streaming potential	2.41	3.46	4.63
Rheology	2.01	3.52	4.45
ITC	1.71	2.60	4.55

### Effect of the Polysaccharide Concentration on Formed Particles

DLS and rheological studies were performed by using different polysaccharide concentrations. Figure S22A represents that higher HyA concentrations correspond to larger particle sizes, since e.g., at *m*_Tr_/*m*_HyA_ = 2 mass ratio, the average hydrodynamic diameter is 263 nm at 0.05 mg/mL and 282 nm at 0.10 mg/mL HyA concentration. The size increase is even more well-marked before the aggregation: 330 nm for the lower and 464 nm for the higher HyA concentration. Also, at higher HyA concentrations, the aggregation occurs at a slightly lower mass ratio (4.20 at 0.01 mg/mL and 4.60 at 0.05 mg/mL). When the rheological behavior of the system is examined (Figure S22B), obvious results were obtained: increasing the HyA concentration creates a much more viscous medium; however, the extent of the decrease in the viscosity is much more significant upon the addition of a given amount of Tr. Also, the breaking point of the curve happens at a smaller *m*_Tr_/*m*_HyA_ mass ratio (4.28 at 0.01 mg/mL and 4.45 at 0.05 mg/mL), as in the case of DLS measurements. These phenomena result from the higher local polymer concentration, as more HyA can form larger aggregates with a given amount of Tr than at lower concentrations.

### Effect of the Experimental Parameters on the Colloidal Dispersions

Based on our previous investigations,^23^ we chose an *m*_Tr_/*m*_HyA_= 2 mass ratio as an optimal composition, since at this point, we can create stable colloidal particles without the risk of rapid aggregation due to an excessive amount of Tr present in the sample. As a first step, we carried out measurements to examine how the dosage speed of the Tr solution influences the size and ζ-potential of the particles (Figure S23A). The results showed that the addition of Tr in one portion (0 s/10 μL) to the HyA resulted in the formation of slightly higher hydrodynamic diameter values (*d* = 290 nm). In the case of slower titrations, neither the size (∼275 nm) nor the ζ-potential (∼−32 mV) varied significantly. Thus, a 20 s/10 μL dosage speed was chosen as the optimal value.

As the next step, the stirring time after preparation was examined. Right after the addition of the last portion of the Tr solution, the turbidity of the dispersion was monitored, as Figure S23B illustrates. The initial increase in turbidity (from ∼38 NTU to ∼100 NTU after 1 h) can be attributed to the continuous formation of colloidal complexes. Since the opalescence of the system does not increase significantly after 1 h, this stirring time was set as the optimal value for the subsequent measurements.

Finally, since the formation of these colloidal systems is mostly based on electrostatic forces, the addition of an inert salt (NaCl) to the particles was examined. Figure S23C and D represent the changes in hydrodynamic diameter, ζ-potential, and turbidity of the system upon increasing the NaCl concentration in the dispersions. The increase in hydrodynamic diameter from 263 to 321 nm (at 0 mM and 20 mM NaCl) is in good agreement with the DLVO theory, which states that the presence of salt in the medium shields the electrostatic repulsion between the particles and initiates aggregation. The shielding effect can also be observed in the increase in ζ-potential values (from −38 mV at 0 mM to −11 mV at 40 mM). Complete aggregation occurs at 30 mM salt concentration, as indicated by a breaking point in the turbidity curve. Due to the more intense aggregation of the particles, the additional NaCl concentration (besides the original amount of NaCl in the buffer medium) was chosen to be 0 mM. The ionic strength-dependent studies revealed that the created particles cannot endure physiological conditions; however, the disintegration of the formulation can possibly lead to, e.g., the release of encapsulated drugs.

### Structural Characterization of the Particles Prepared at *m*_Tr_/*m*_HyA_= 2

After a thorough examination of the pH, concentration, and experimental parameters, PEC particles with optimal parameters were successfully created. For their characterization, FT-IR and CD spectroscopy, TEM, and DSC measurements were carried out (Figure 5). Based on the FT-IR and CD studies, changes in the secondary structure of the protein can be observed, indicating the formation of PEC-based colloidal particles instead of the existence of a physical mixture of the macromolecules. According to the FT-IR spectra (Figure 5A), the signals of the protein dominate in the spectrum of the complexes, which may suggest that the more significant part of the particles is Tr, aligning well with the Tr:HyA 2:1 mass ratio. Moreover, the O–H bond stretch at 3279 cm^–1^ resembles that of Tr, and the amide peaks (1635 and 1528 cm^–1^) of the protein are dominant in the spectrum of the complex. For the determination of the secondary structure of the protein and the complexes, the amide I. peak (1580–1730 cm^–1^) was deconvoluted in both cases (Figure S24).^33^ As shown in Table 2, the α-helix structure of the protein decreases (from 8.20 to 1.90%) when the protein is in the particles; however, the β-sheet content increases (from 36.4 to 42.4%), while the random and turn content of Tr do not change significantly. The CD spectrum (Figure 5B) of the free protein in the pH 4.5 buffer differs from that of the Tr in the particles (Table 2).

**Figure 5:**
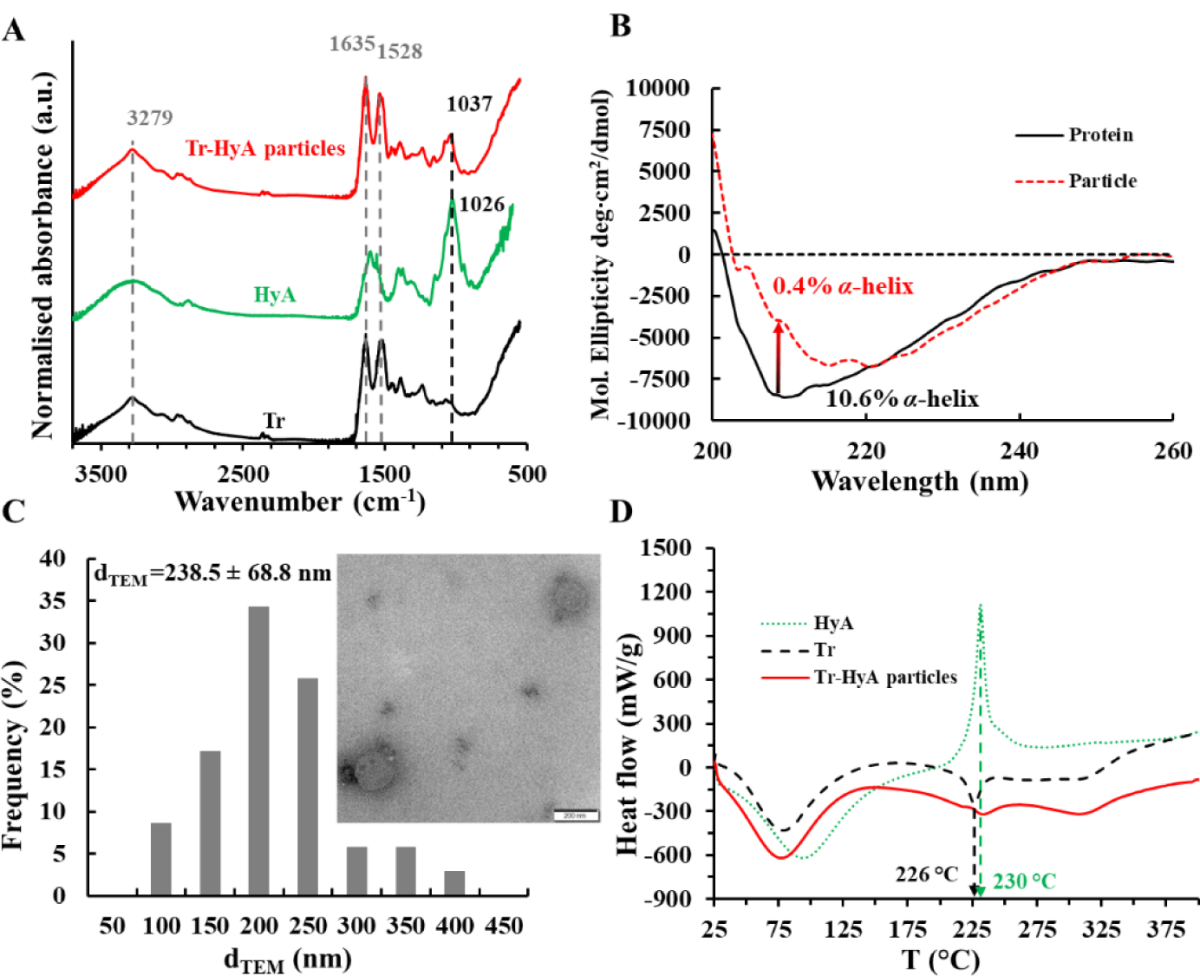
(**A**) FT-IR spectra of Tr, HyA, and Tr-HyA particles (solid samples, *m*_Tr_/*m*_HyA_ = 2) and (**B**) CD spectra of the particles and Tr (*m*_Tr_/*m*_HyA_ = 2, *c*_HyA in sample_ = 0.01 mg/mL, *c*_Tr in sample_= 0.02 mg/mL). (**C**) Size distribution of the particles by TEM with a representative image (*m*_Tr_/*m*_HyA_ = 2) and (**D**) DSC curves of Tr, HyA, and Tr-HyA particles (solid samples, *m*_Tr_/*m*_HyA_ = 2).

**Table 2:** Percentual Distribution of Secondary Structure of Tr in Its Free Form and in Tr-HyA Particles Obtained by CD (*c*_Tr_ = 0.02 mg/mL) and IR (Solid Powder with Same pH)

	Tr		Tr-HyA	
	CD	IR	CD	IR
α-helix	10.6	8.20	0.40	1.90
β-sheet	47.5	36.4	66.3	42.4
Turn	4.2	23.8	0.0	24.2
Random	37.7	31.6	33.3	31.5
Total	100	100	100	100
RMS value	10.812		22.301	

According to Reed’s model, the α-helix content of the free protein is 10.6%, for β-sheet this value is 47.5%. The ratio of the secondary structures compared to each other resembles ref 34. When Tr is in the particles, the α-helix content decreases to 0.4%, and the β-sheet content changes to 66.3%, which is a trend similar to the results obtained by IR spectroscopy. Also, this trend can be explained by the shape of the spectra, as the minimum of the free protein is shifted toward higher wavelengths when in the particles, which corresponds to the absorbance regime of the β-sheet structure.^35^ In summary, CD spectra confirm that when the interaction happens between the macromolecules, the protein loses its α-helix content which can be explained by the unfolding of the protein. This enables the positive charges of the protein to bind to the negative charges of HyA, thus, creating colloidal particles. Also, the β-sheet content increases, which is a sign of denaturation or aggregation upon interacting with the polysaccharide, which further confirms the creation of water-insoluble particles. When the secondary structure of a protein changes significantly, its originally hydrophobic regions can become available, which can be advantageous for the encapsulation of poorly water-soluble drug molecules through hydrophobic interactions. These changes of the secondary structure of the protein can lead to significant changes in the biological functions of Tr;^36^ however, if the primary goal is the creation of stable colloidal particles, then the loss of the biological function of the protein is not as essential.

To confirm the validity of the DLS measurements, TEM images were taken of the particles. Since the medium contained buffer salt in such a quantity that the salt crystals made taking TEM images difficult, dialysis was carried out to eliminate most of the salt from the medium. The optimal dialysis time was found to be 1 h, as shown in Figure S25A, by measuring the specific conductance of the solution outside the membrane. The size of the particles was determined after dialysis by DLS and was found to be 253 nm. This indicated that this step did not have a significant effect on the particles. By examining the dispersion with TEM, the average particle size was found to be 238 nm (Figure 5C); however, there are 2 main problems with taking images of them. The first one is that these are very soft particles, making it difficult to achieve enough contrast compared to the background. This is why the images of the particles are noisy. The second problem, which makes it hard to take images of them, is that the electron beam can burn the samples quite quickly, making focusing even harder. The difference from the hydrodynamic diameter obtained by DLS is due to the lack of a hydrate shell, drying of the sample, the lack of contrast, which made finding the edge of the particles hard, and the fact that with TEM, a number-weighted size distribution can be calculated, while with DLS, intensity-weighted values are obtained.

Finally, thermoanalytical analysis of the particles was carried out (Figure 5D). Similarly to the FT-IR measurements, the solid HyA, Tr, and their complex were investigated with DSC. The curves show a sharp exothermic peak for HyA at 230 °C and an endothermic peak for Tr at 226 °C. In contrast, in the curve of the Tr-HyA complex, both sharp peaks disappear, which may indicate interaction between the macromolecules. Moreover, since the DSC curve of the particles resembles that of the pure protein much more closely, we can conclude that the formulation is rich in Tr, which corresponds well with the IR spectroscopic results.

To further examine the characteristics of the particles, they were compared to one of our former systems made of HyA and HSA.^23^ Particles at the *m*_protein_/*m*_HyA_= 2 mass ratio were prepared for the HSA-HyA and Tr-HyA systems, and the stability of the complexes was examined over time. According to Figure 6 the samples containing HSA had a lower hydrodynamic diameter in the first few days of the examination (243 nm), which is in good agreement with our former findings. However, after the second day, a rise in the size of the particles can be seen, which is followed by the aggregation of the complexes after 8 days (605 nm). In contrast, the Tr-HyA particles have a greater size at the beginning (270 nm), but a much slower increase in the hydrodynamic diameter can be seen even after 13 days (368 nm). These measurements can be promising from the point of view of many application fields in which the stability of formulations must be well-controlled.

**Figure 6:**
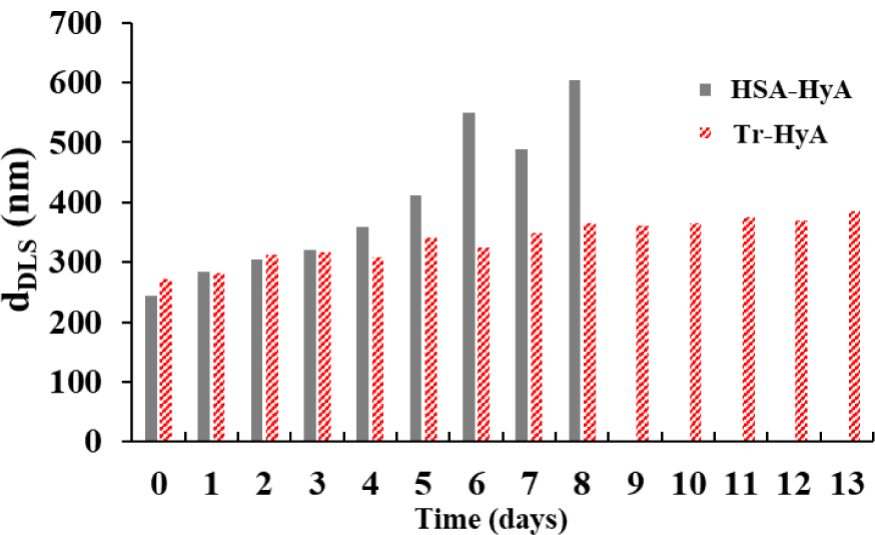
Change in the diameter of the HSA-HyA and the Tr-HyA particles at pH = 4.5 as a function of storage days (*m*_protein_/*m*_HyA_= 2).

### Encapsulation of Different Water-Insoluble Compounds

Although the main scope of this article was to study the self-assembly of macromolecules, the encapsulation possibilities of different extremely hydrophobic drugs were also tested. Based on this, we selected compounds (Vitamin D3, Vitamin K1, curcumin, quercetin, and artesunate) that have relatively similar molar masses (∼300–450 Da). The encapsulation of these drugs was executed at 3 different concentrations, which was followed by determination of the light scattering features (hydrodynamic size and ζ-potential) of the systems; the encapsulation efficiency (EE%) and drug loading capacity (DL%) of the particles were calculated in every case, as shown in Table 3. In the case of Vitamins K1 and D3, high EE% (79–84% and 72–93%, respectively) and DL% values were observed, successfully proving that most of the vitamins can be encapsulated into the particles. This fact is further supported by the significant increase in the hydrodynamic size of the particles (309–407 nm and 327–405 nm, respectively) in both cases, along with good polydispersity (PDI) values (except for K1 at the highest concentration), and the ζ-potential of the particles decreased. TEM images (Figure S26) of Tr-HyA-K1 and Tr-HyA-D3 particles indicate that the drug can be found on the surface of the particles; however, in the case of K1, the drying process resulted in vitamin liquefying (since it is an oily material), which worsened the quality of the images. For this reason, no further evaluation of the TEM images is presented Using nanoformulation, the water solubility of D3 was increased from a concentration of 58.8 μM to 188 μM at the highest drug concentration, representing more than a threefold increase in solubility. For K1, the scientific literature provides very limited data regarding its solubility in aqueous media , often referring to it simply as “insoluble”. This highlights the importance of our work, as we K1 concentration of 80.2 μg/mL (178.2 μM). In the case of the Cur and Que-containing particles, the polydispersity of the samples rose above 0.3, and the hydrodynamic diameters increased significantly. For Que, this resulted in complete aggregation of the system at the highest drug content. Moreover, very low drug content was detected in these particles, suggesting that these molecules cannot be encapsulated by the Tr-HyA particles.

**Table 3:**
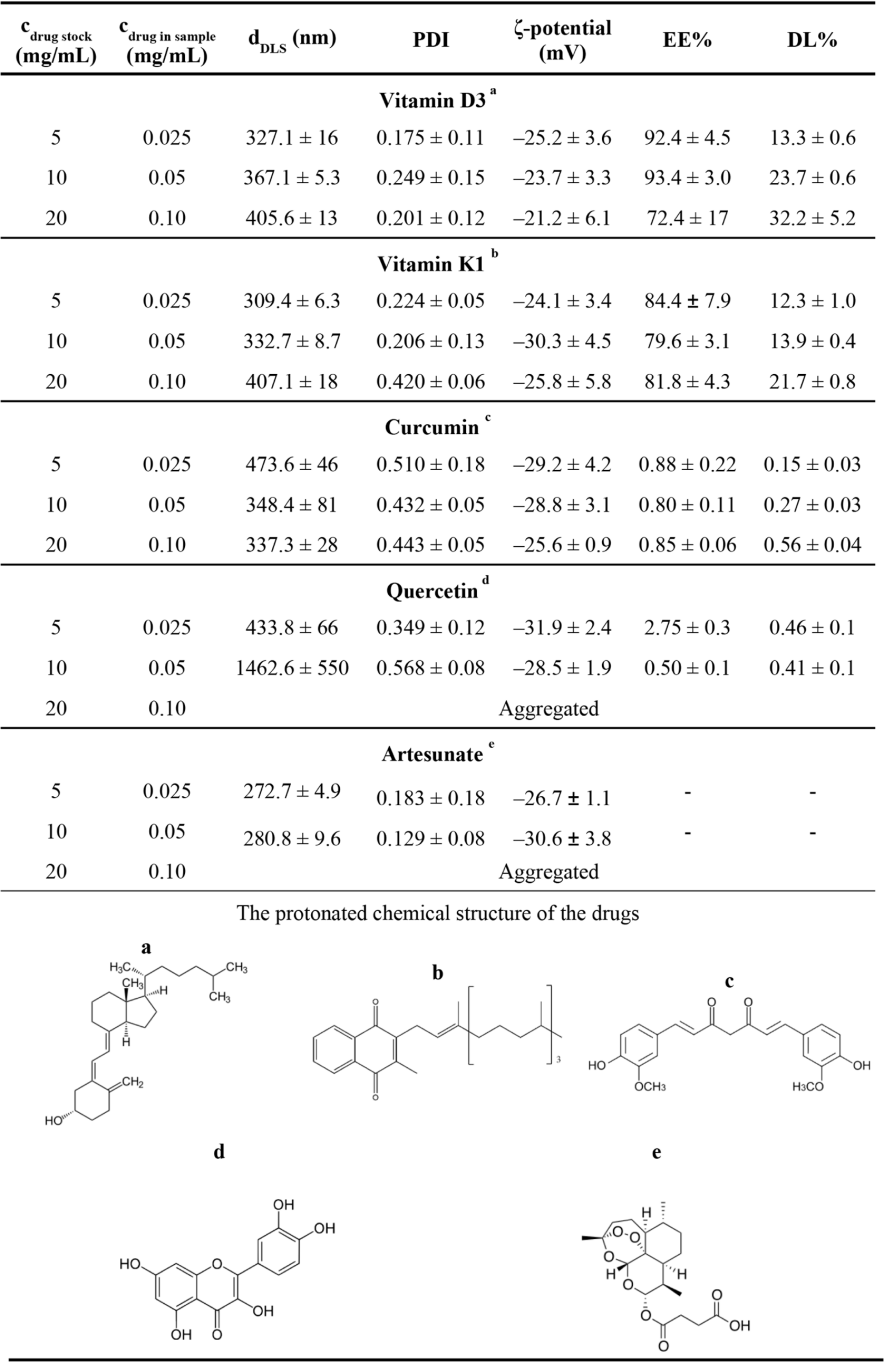
Particle Diameter, PDI, ζ-Potential, EE%, and DL% of Tr-HyA Particles with the Protonated Chemical Structure of the Drugs

The main difference between the EE% values of the vitamins and the Que-Cur systems might be due to the rate of crystallization of the drugs in aquatic environments. Since K1 and D3 have amphiphilic structures, they can create smaller particles in the acetate buffer too (according to our DLS measurements, 231.6 ± 6.4 and 178.8 ± 5.5 nm, respectively), which prevents them from sedimentation immediately after being injected into the aquatic environment. This way, they can participate in the particle formation when Tr and HyA are present in the system, resulting in the formation of larger particle sizes (300–420 nm) compared to the unloaded Tr-HyA particles (263 nm).

Regarding Art, lower particle sizes (270–280 nm) could be detected; however, the ζ-potential of the particles was similar to those measured during the encapsulation of other drugs. When it came to the EE% and DL% determination, UV–vis spectroscopy could not be used since no characteristic peak appeared in the spectrum. To overcome this problem, calibration curves were measured for Art using DSC, as shown in Figure S14. During the encapsulation, the highest Art concentration caused the particles to aggregate, and in the case of lower drug concentrations, the characteristic exothermic peak of Art did not appear in the DSC thermograms (Figure S27) at all, which indicated that the encapsulation was not successful.

The explanation for the encapsulation of K1 and D3 besides the lack of sedimentation, lies in the fact that although the individual Tr and HyA are rather hydrophilic, due to the self-assembly process, an interface can be formed. As a result, the hydrophobic drug molecules will prefer encapsulation and adsorption from the polar water solvent onto the surface of macromolecular chains, which further facilitates the formulation.

A further comparison to our former findings (HSA-HyA system^23^) was carried out regarding the encapsulation of vitamin D3. Since the D3-containing Tr-HyA particles were prepared at the same experimental conditions as the HSA-HyA particles, we had the opportunity to compare them. In the case of Tr-HyA-D3 particles, the presence of the vitamin increases the size of the particles significantly (to 320–400 nm) and lowers the ζ-potential of the complexes compared to the unloaded ones; however, in the case of HSA-HyA particles this phenomenon occurs to a lesser extent (240–270 nm). When examining the amount of vitamin encapsulated by the Tr-HyA particles, we can see that the encapsulation efficiency (EE%) is almost the same in the first two cases (∼92%) and lower in the third one (72.4%); however, the drug loading values increase monotonously (from 13% to 32%). For the HSA-HyA particles, the EE% (43–69%) and the drug loading values (10–22%) are smaller. This greater encapsulated drug amount is further confirmed by the higher hydrodynamic size values for the Tr-HyA system.

As a summary, in the case of both proteins, D3 tends to enrich in the particles due to its hydrophobic character, which provides a more hydrophobic environment than the aqueous phase. This can explain the high EE% values. It can also be concluded that our new PEC-type colloidal formulation is capable of encapsulating hydrophobic substances, showing promising potential as a drug delivery vehicle. The size of the formulation is relatively large for the penetration of drugs across the blood–brain barrier (BBB) (∼100–200 nm) or nasal administration (∼300 nm), but its application as a dietary supplement or use as carrier particles for a controlled release system in the vaginal environment is suitable.

### Drug Dissolution Studies

After the encapsulation of K1 and D3, the dissolution profiles (Figure 7) of the pure drugs (•) were compared to the formulated ones (▲). All dissolution studies were carried out in PBS buffer; however, due to the poor water solubility of these compounds, an additive was needed to be added to the buffer. This allowed the drugs to be solubilized, and thus the calibration curves (Figures S15 and S16) of the compounds could be created, enabling us to observe the differences in the dissolution profiles between the formulated and unformulated drugs. For this, ethanol–PBS solvent mixtures with different compositions were made; however at low ethanol contents, the drugs did not dissolve, and at high ethanol contents, the buffer salt precipitated, which would have made spectrophotometric measurements unfeasible. Therefore, different surfactants (sodium dodecyl sulfate (SDS), Tween 80, cetyltrimethylammonium bromide (CTAB), pluronic (PLUR), tocopherol polyethylene glycol succinate (TPGS), and Triton X-100 (TX-100)) were tested for this purpose. Most of them could solubilize D3; however, only TX-100 was able to keep K1 in solution, which is why we chose this surfactant as an additive to PBS. This phenomenon might be due to the similarity in the chemical structure of TX-100 and K1, as both have an aromatic ring and a hydrophobic tail, which could interact synergistically.

**Figure 7:**
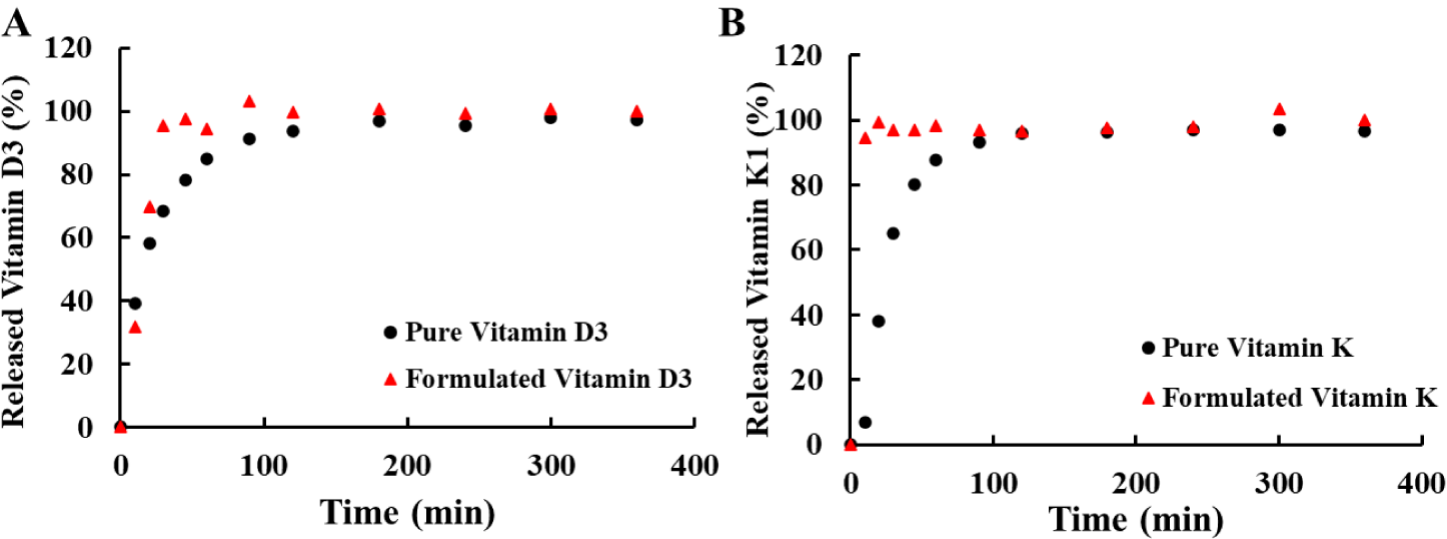
Experimental dissolution data of (**A**) pure and formulated D3 and (**B**) pure and K1-containing colloidal carriers at pH = 7.4 (PBS buffer with 5 mg/mL of TX-100) at 37 °C.

Based on the experimental data from the dissolution studies for both K1 and D3, we can clearly see that the formulation enhances the release of the drugs: the release curves reach saturation much faster when the drug is formulated into the particles. Also, we can conclude that after 120 min, 93.7% of the pure D3 is dissolved in the medium (Figure 7A), whereas in the case of the formulated drug, only 30 min are needed to reach 95.3% of the released drug amount. A similar but much faster phenomenon can be seen in the case of K1 (Figure 7B), as 94.7% of the formulated drug is dissolved in the medium in only 10 min, while for the unformulated drug, 90 min are required to achieve 93.1% liberated drug content.

The explanation of this rapid dissolution can be approached from two sides: one is from the side of the physical form of the pure drugs, and the other is from the disintegration of the carrier particles. However, the D3 is a solid powder; it has a relatively small specific surface (compared to nano/colloidal carriers. The same problem occurs when it comes to the release of K1, with the difference being that it is a viscous liquid. When the colloidal carriers are introduced into the medium, after hydration, the complexes can fall apart, resulting in a burst release of the drug. This structural disintegration can also be seen when the unloaded particles are dialyzed against SIF, SGF, and PBS, as the structure of the carrier is broken down (Table S1), meaning that the DLS cannot determine the well-defined hydrodynamic size and PDI value of the particles, which were measured after the preparation.

## Conclusions

Polyelectrolyte complexes containing oppositely charged macromolecules, such as human apo-transferrin protein and high-molecular-weight hyaluronic acid polysaccharide, were fabricated by a simple self-assembly process. This article is the first to quantitatively interpret the formation process of these protein–polysaccharide complex colloidal particles using experimental results from several physicochemical and colloid chemistry techniques, including light scattering, rheology, titration microcalorimetry, and electron microscopy. The structural change of the protein was monitored through infrared and circular dichroism spectroscopic studies. It was concluded that the pH and mass ratio of the macromolecules greatly influence the self-assembly process. Particles with enhanced stability were prepared at transferrin–hyaluronic acid = 2:1 mass ratio, where the particle size of 240–260 nm was confirmed. The applicability of these colloid particles as drug delivery systems was proved for cholecalciferol (Vitamin D3) and Vitamin K1. The water solubility was increased 3 times for both cases (assuming similar water solubility), and drug dissolution was also improved. Finally, it can be stated that the change of the protein from HSA to Tr not only improves the drug-loading ability of the particles but also increases their long-term stability, which can be further enhanced by using gastroprotective capsules or gel matrices for further applications in physiological environments.

## Supplementary Material



## Notes

Interpretation of size distribution functions of the created particles at different pH; photos of the samples; the effect of accumulation time during the DLS measurements on the correlation and the size distribution functions; correlation functions (by DLS); measured and fitted ζ-potential distributions; CD spectra; UV–vis calibration curves for drugs; calibration DSC thermograms; UV–vis spectra; polydispersity indices; ITC enthalpograms; rheological titration curves; deconvoluted FT-IR spectra; TEM images; DSC curves; stability data (PDF)
