# Supplementary material for: Detailed Physicochemical Analysis of Hyaluronic Acid and Transferrin Self-Assembly To Produce Drug Carrier Colloids
Source: ACS Omega. 2025 Nov 11;10(46):56520–32. doi: 10.1021/acsomega.5c08615 (PMC12658641; doi:10.1021/acsomega.5c08615)
Supplement: Supplementary file 1 [file ao5c08615_si_001.pdf]

Detailed Physico-Chemical Analysis of Hyaluronic Acid and Transferrin Self-Assembly to Produce Drug Carrier Colloids

László Seres[1,2], Norbert Varga[1,2], Bianka Torma[1,2], Ádám Juhász[1,2], Edit Csapó[1,2,*]

[1] Interdisciplinary Excellence Center, Department of Physical Chemistry and Materials Science, University of Szeged, H-6720 Rerrich B. sqr. 1, Szeged, Hungary

[2] MTA-SZTE Lendület "Momentum" Noble Metal Nanostructures Research Group, University of Szeged, H-6720 Rerrich B. sqr. 1, Szeged, Hungary

* Corresponding author: Edit Csapó (juhaszne.csapo.edit@med.u-szeged.hu)

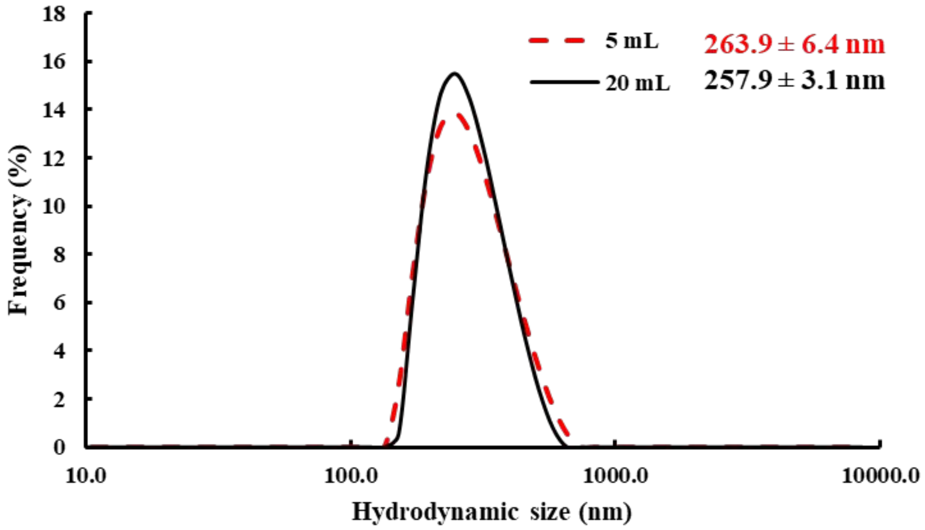

Figure S1. Representative size distribution functions of the created particles when prepared in 5 or 20 mL portions (c_{HyA}= 0.05 mg/mL, $c_{Tr\ titrating}$= 5 mg/mL)

A B C

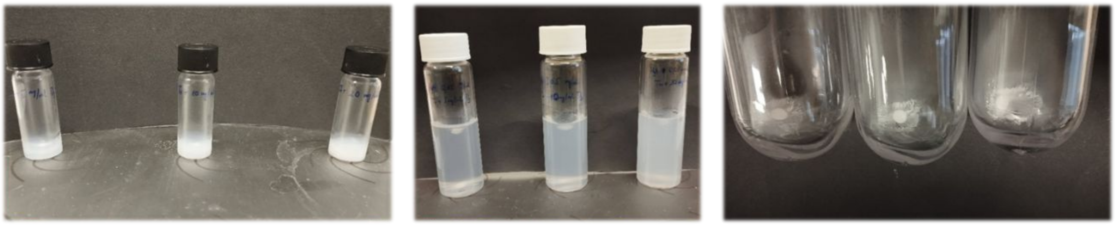

Tr-D3

D3 stock solution concentrations from left to right:
5, 10, 20 mg/mL

HyA-Tr-D3

D3 stock solution concentrations
from left to right:
5, 10, 20 mg/mL

HyA-Tr-D3 after centrifugation and lyophilization

D3 stock solution concentrations from left to right:
5, 10, 20 mg/mL

Figure S2. Photos of (**A**) the Tr-D3 titrating solutions, (**B**) the colloid dispersion of Vitamin D3-containing HyA-Tr particles after preparation and (**C**) the solid powders after centrifugation and lyophilization *(Photos were taken by László Seres first author)*

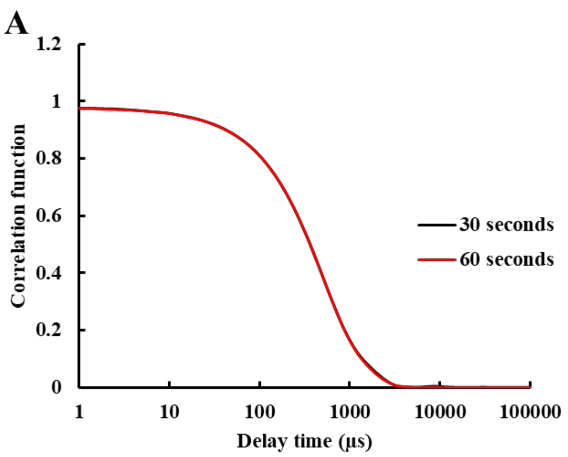

Figure S3. The effect of accumulation time during the DLS measurements on the (**A**) correlation and (**B**) the size distribution functions

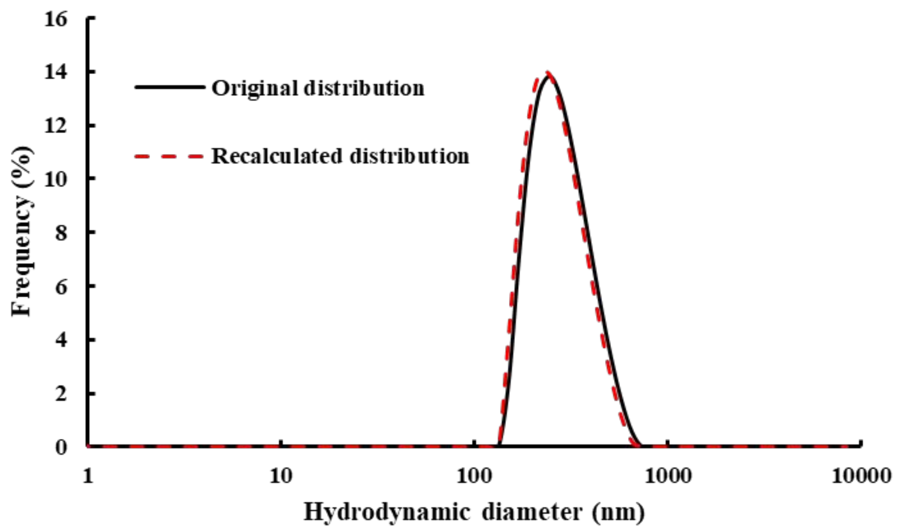

Figure S4. The original (calculated by the viscosity of the buffer) and the recalculated (calculated by the viscosity of the HyA solution) size distribution functions of the Tr-HyA particles at $m_{Tr}/m_{HyA} = 2$

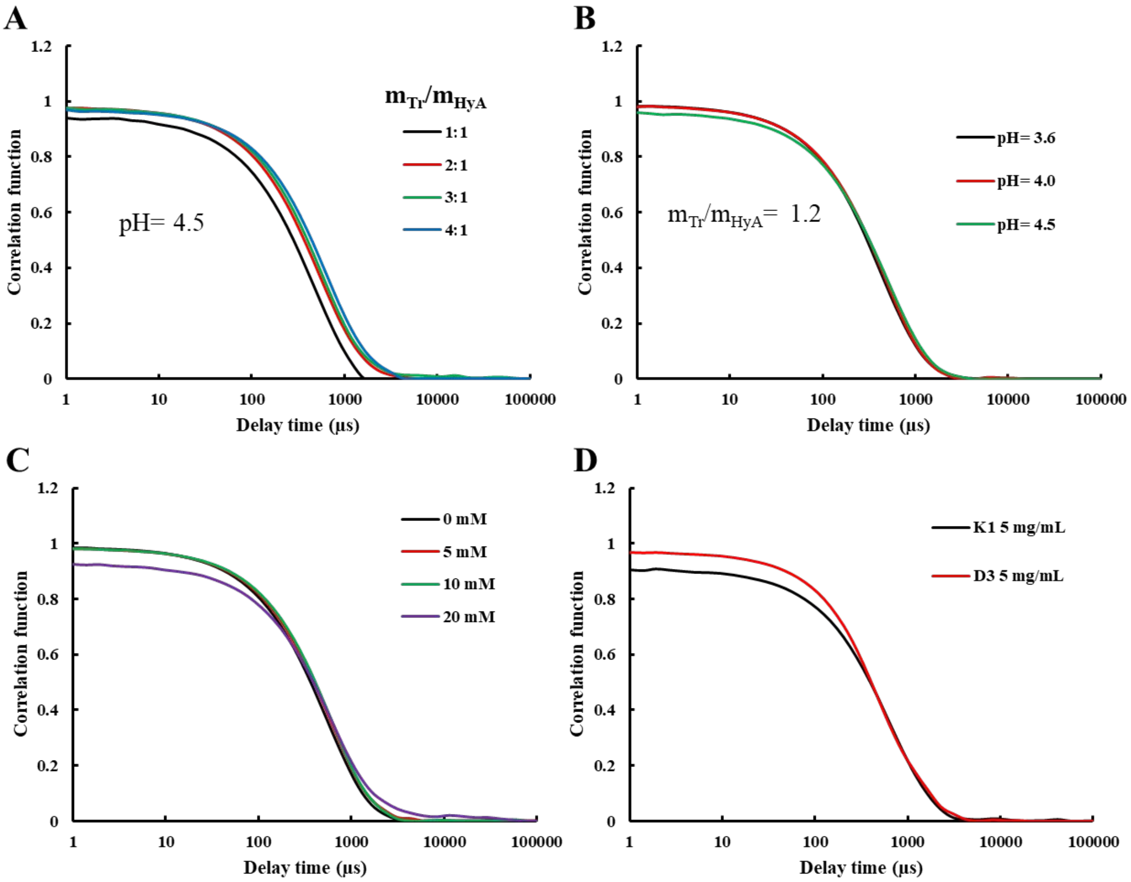

Figure S5. The correlation functions of (**A**) the empty particles at different mass ratios at pH = 4.5, (**B**) at different pH values at m_{Tr}/m_{HyA}= 1.2, (**C**) at different added salt concentrations and (**D**) the Vitamin K1 and D3 loaded particles (c_{D3} and $_{K1}$ ethanolic stock = 5 mg/ml, m_{Tr}/m_{HyA} = 2)

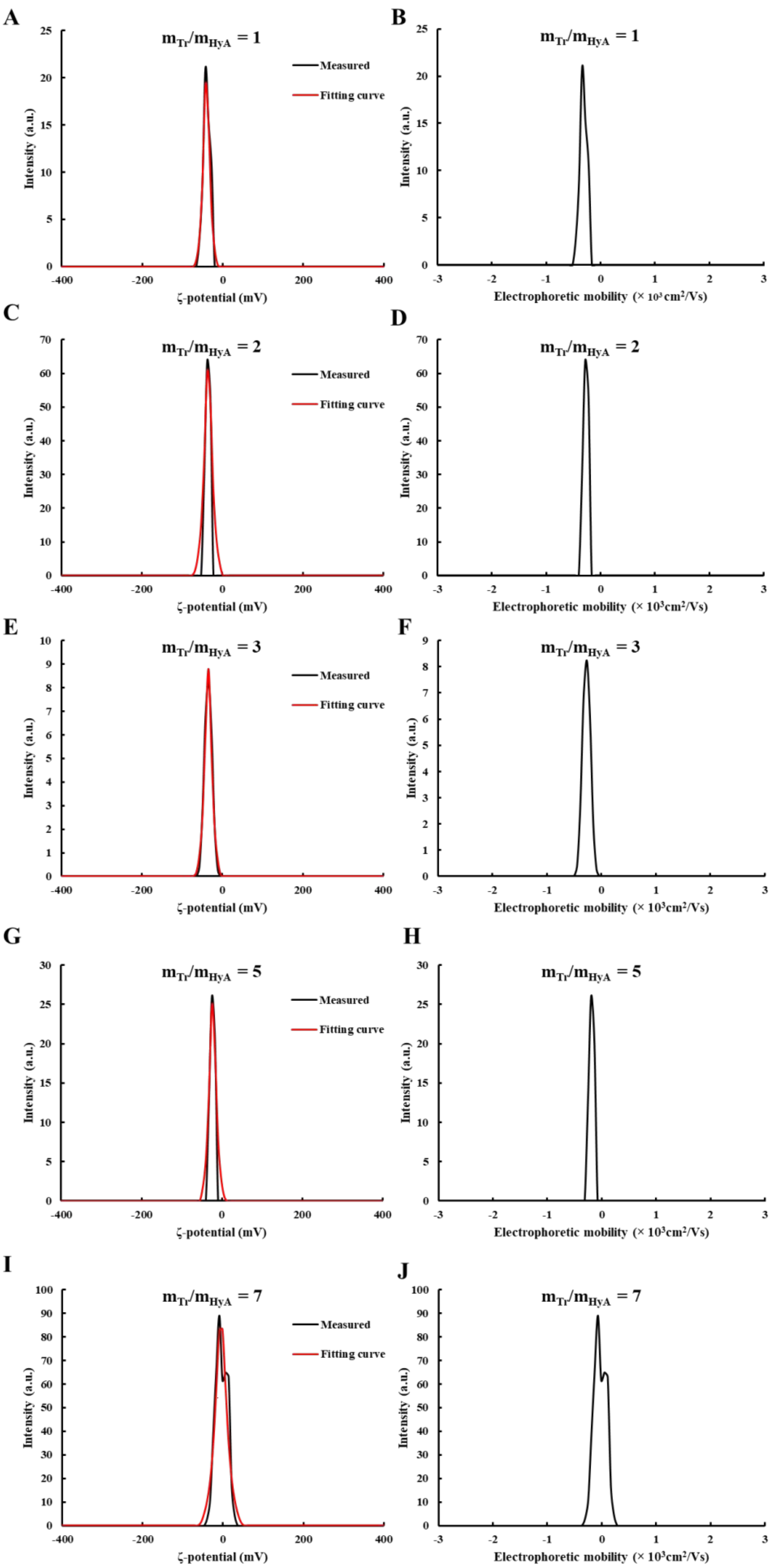

Figure S6. (**A**), (**C**), (**E**), (**G**), (**I**) The measured and fitted ζ-potential distributions of the empty Tr-HyA particles at different mass ratios at pH = 4.5 and (**B**), (**D**), (**F**), (**H**), (**J**) the electrophoretic mobility distributions of the same samples

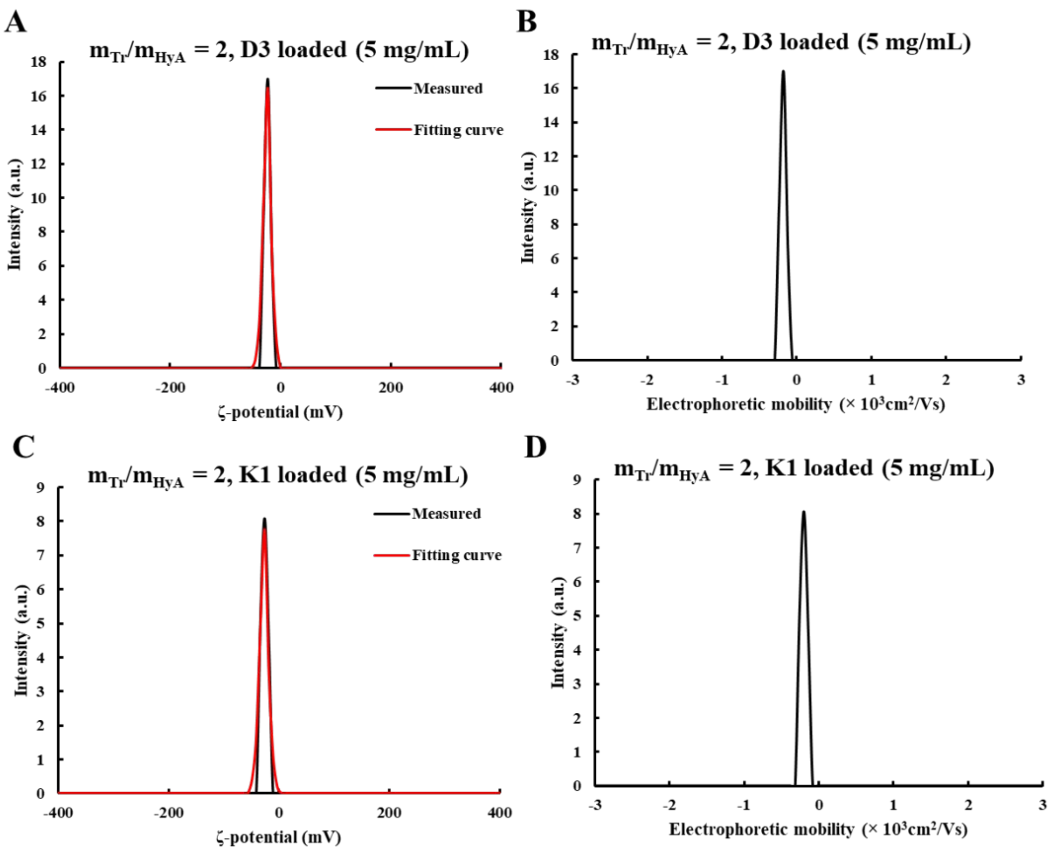

Figure S7. (**A**), (**C**) The measured and fitted ζ-potential distributions of the D3 and K1 loaded Tr-HyA particles (m_{Tr}/m_{HyA}= 2, pH= 4.5) and (**B**), (**D**) the electrophoretic mobility distributions of the same samples

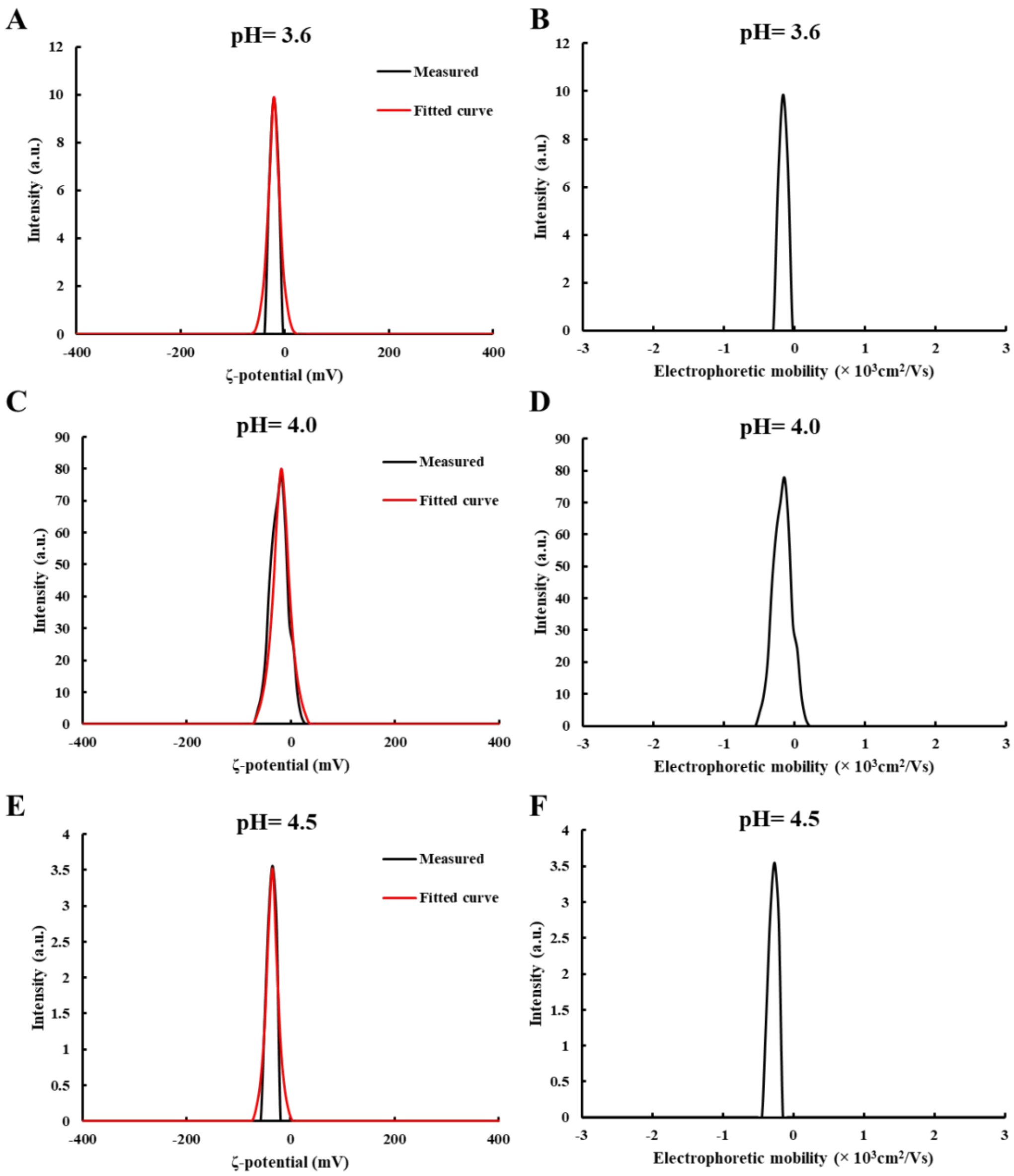

Figure S8. (**A**), (**C**), (**E**) The measured and fitted ζ-potential distributions of the empty Tr-HyA particles at different pH values at $m_{Tr}/m_{HyA} = 2$ and (**B**), (**D**), (**F**) the electrophoretic mobility distributions of the same samples

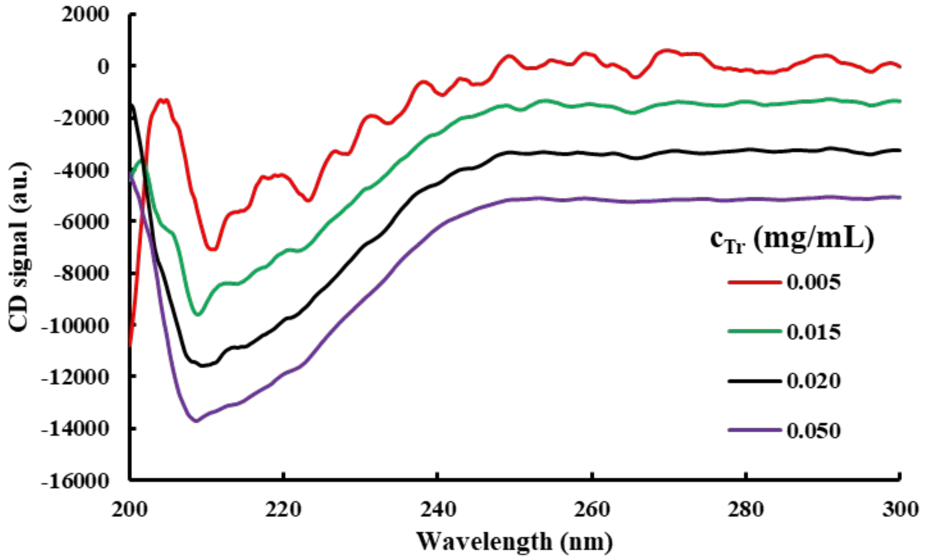

Figure S9. CD spectra of Tr in pH = 4.5 acetate buffer at different concentrations. For the better visibility, the spectra were shifted towards the lower CD signal units (with 1000, 3000 and 5000 units for the 0.015, 0.020, 0.050 mg/mL samples, respectively)

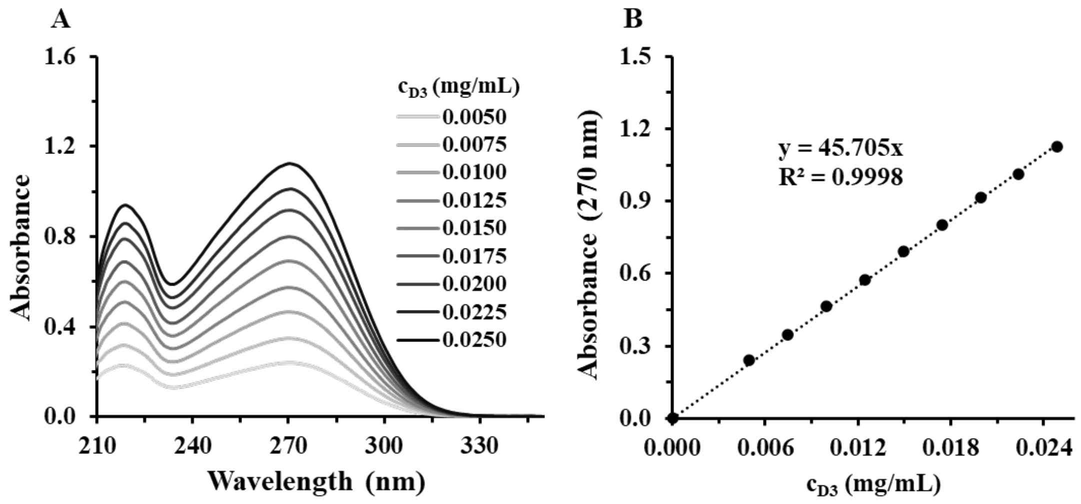

Figure S10. (**A**) The calibration UV-VIS spectra and (**B**) the calibration line of vitamin D3 in ethanol

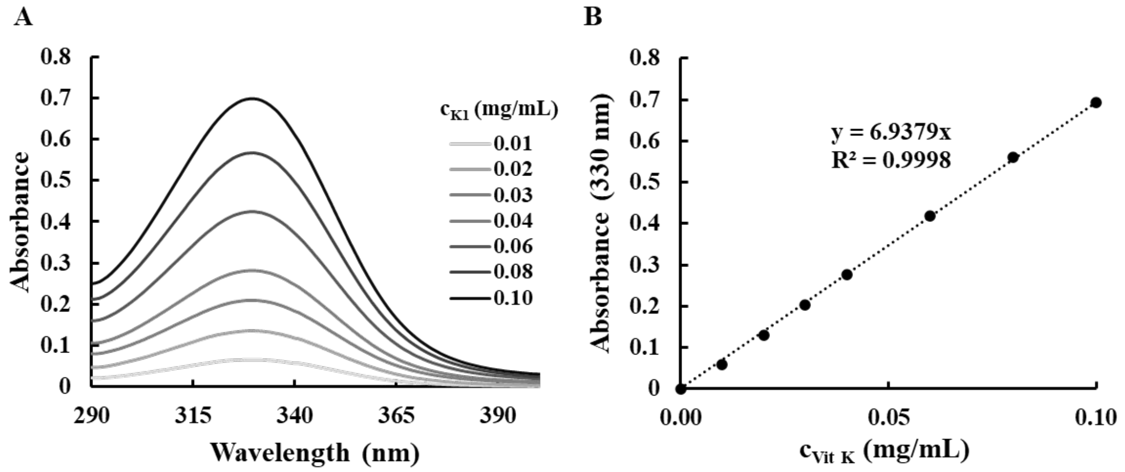

Figure S11. (**A**) The calibration UV-VIS spectra and (**B**) the calibration line of vitamin K1 in ethanol

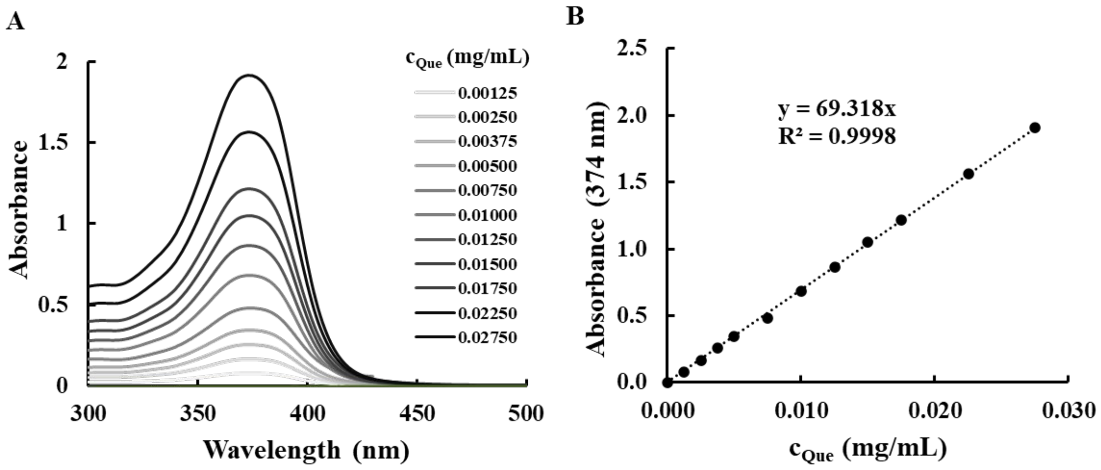

Figure S12. (**A**) The calibration UV-VIS spectra and (**B**) the calibration line of quercetin in ethanol

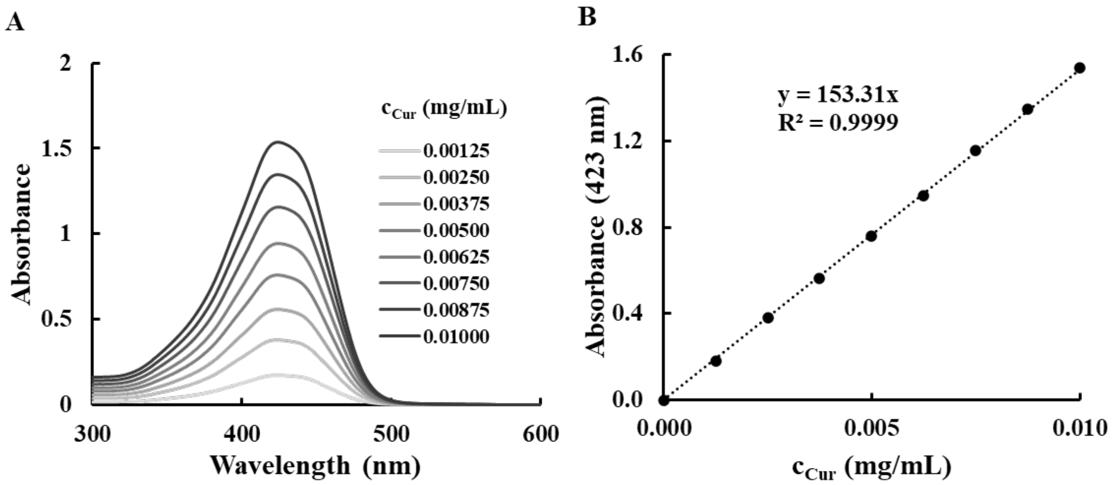

Figure S13. (**A**) The calibration UV-VIS spectra and (**B**) the calibration line of curcumin in ethanol

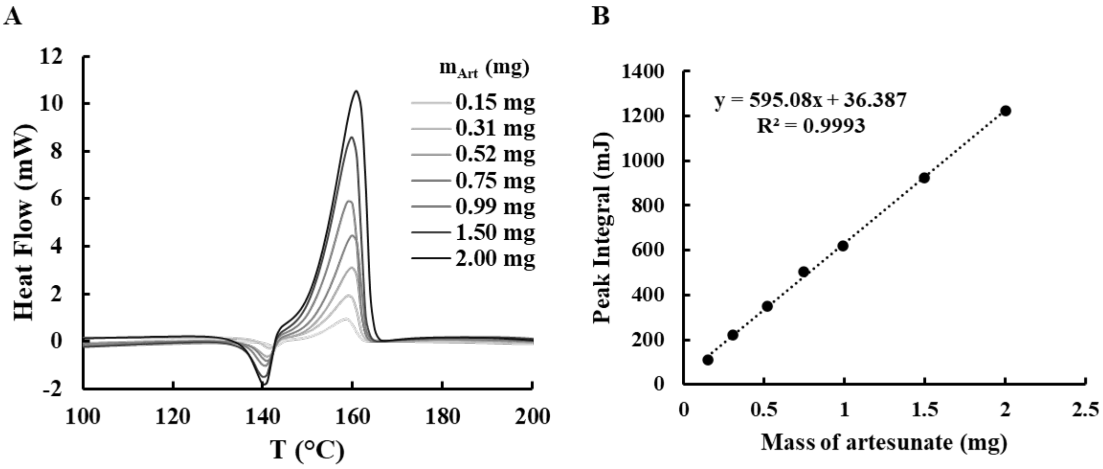

Figure S14. (**A**) The calibration DSC thermograms and (**B**) the calibration line of artesunate

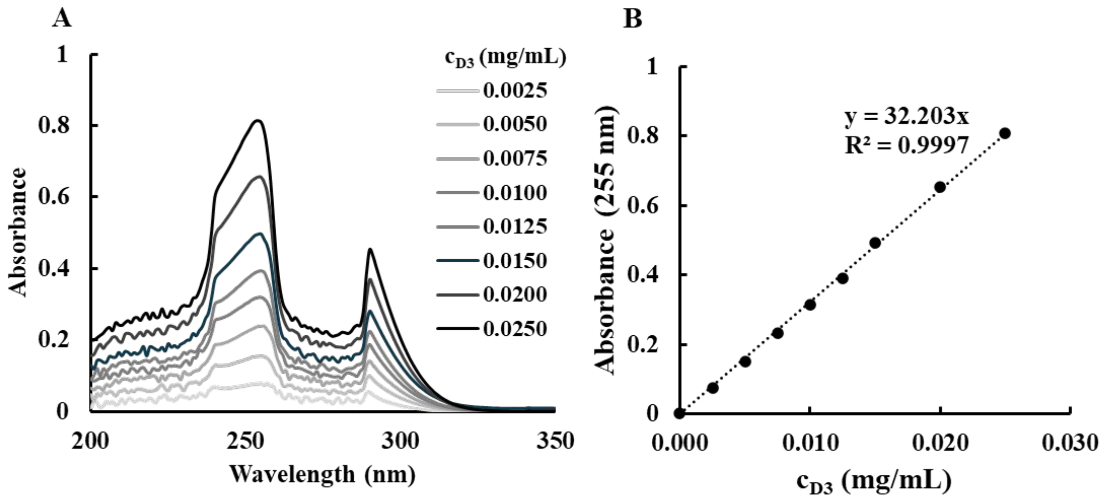

Figure S15. (**A**) The calibration UV-VIS spectra and (**B**) the calibration line of D3 in PBS-TX-100

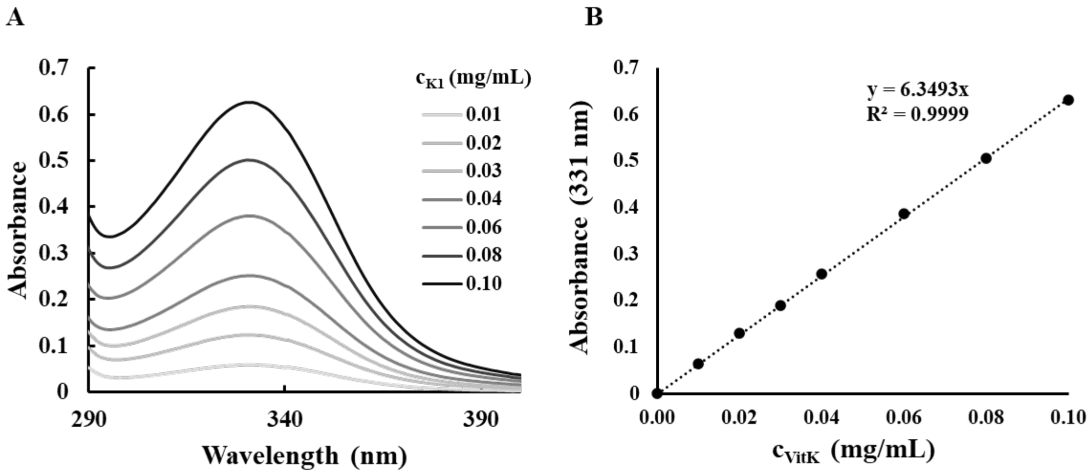

Figure S16. (**A**) The calibration UV-VIS spectra and (**B**) the calibration line of K1 in PBS-TX-100

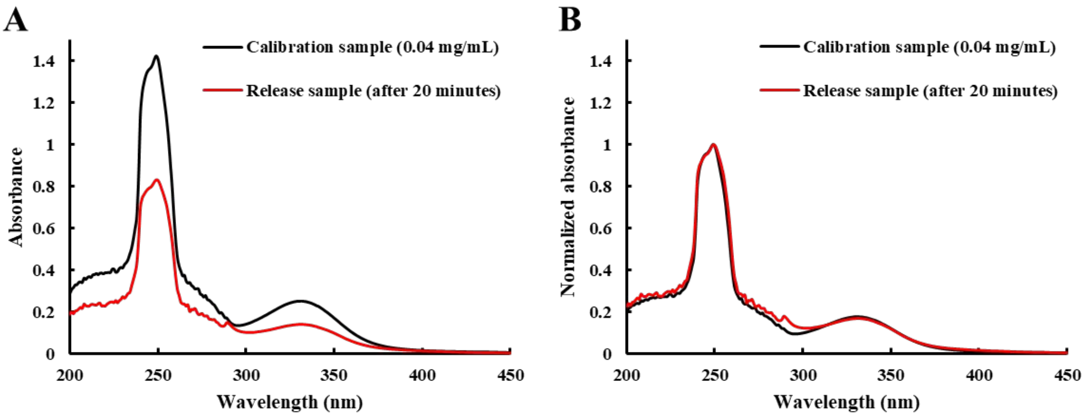

Figure S17. (**A**) The raw and (**B**) normalized UV-VIS spectra of the 0.04 mg/mL calibration curve and the data registered after 20 minutes of release

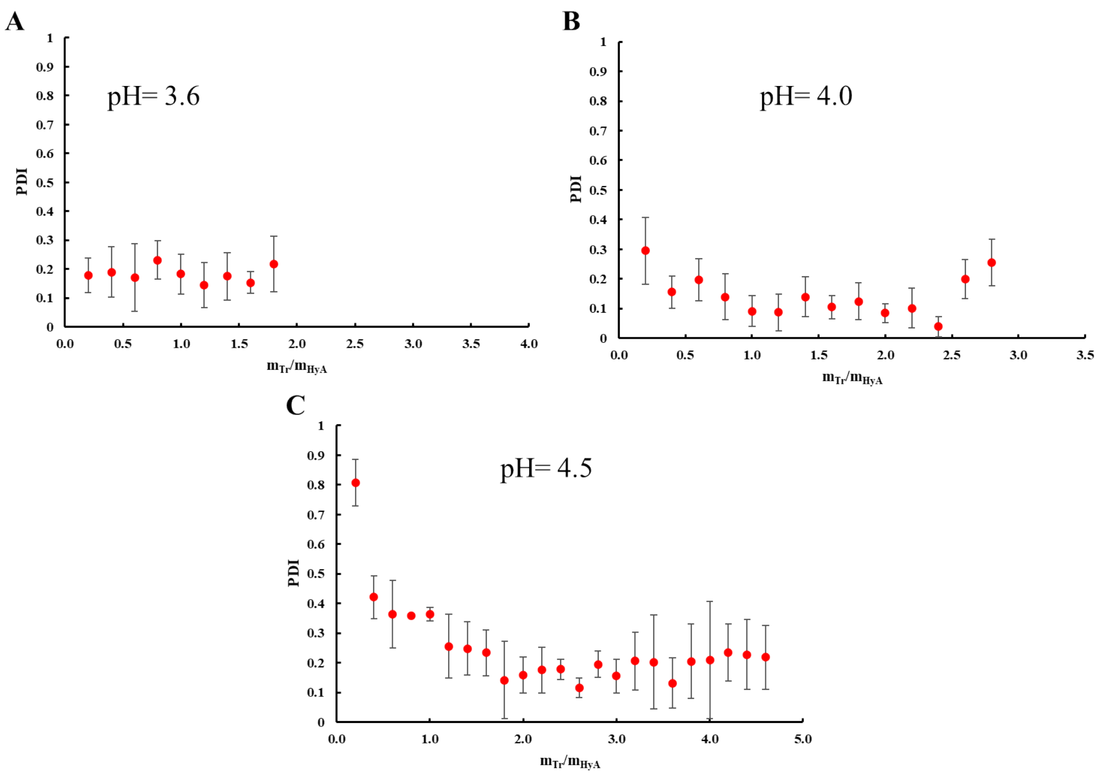

Figure S18. Polydispersity indices (PDI) of the particles during the DLS titrations of HyA with Tr at different pH values: (**A**) pH = 3.6; (**B**) pH = 4.0; (**C**) pH = 4.5 (c_{HyA}= 0.05 mg/mL, $c_{Tr\ titrating}$= 5 mg/mL)

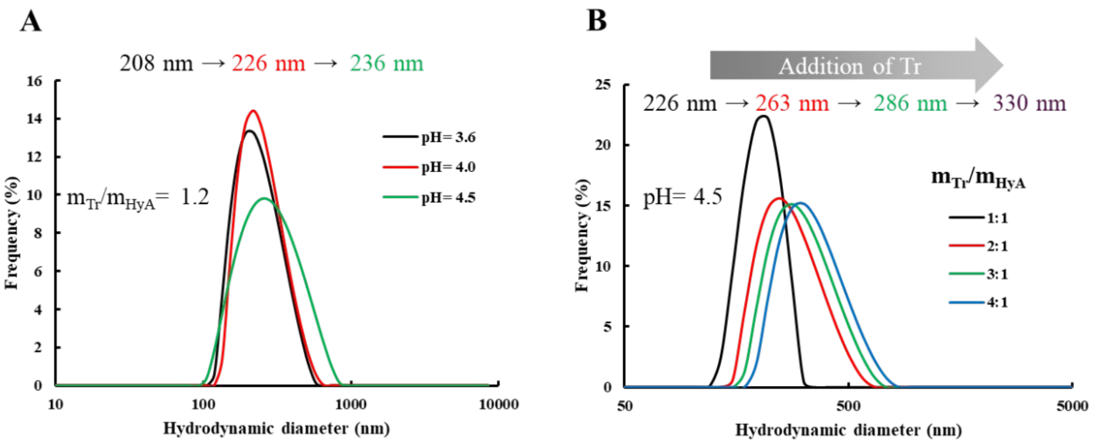

Figure S19. The size distribution functions of Tr-HyA particles during DLS titrations (**A**) at different pH values (m_{Tr}/m_{HyA} = 1.2) and (**B**) at different macromolecule mass ratios (pH= 4.5)

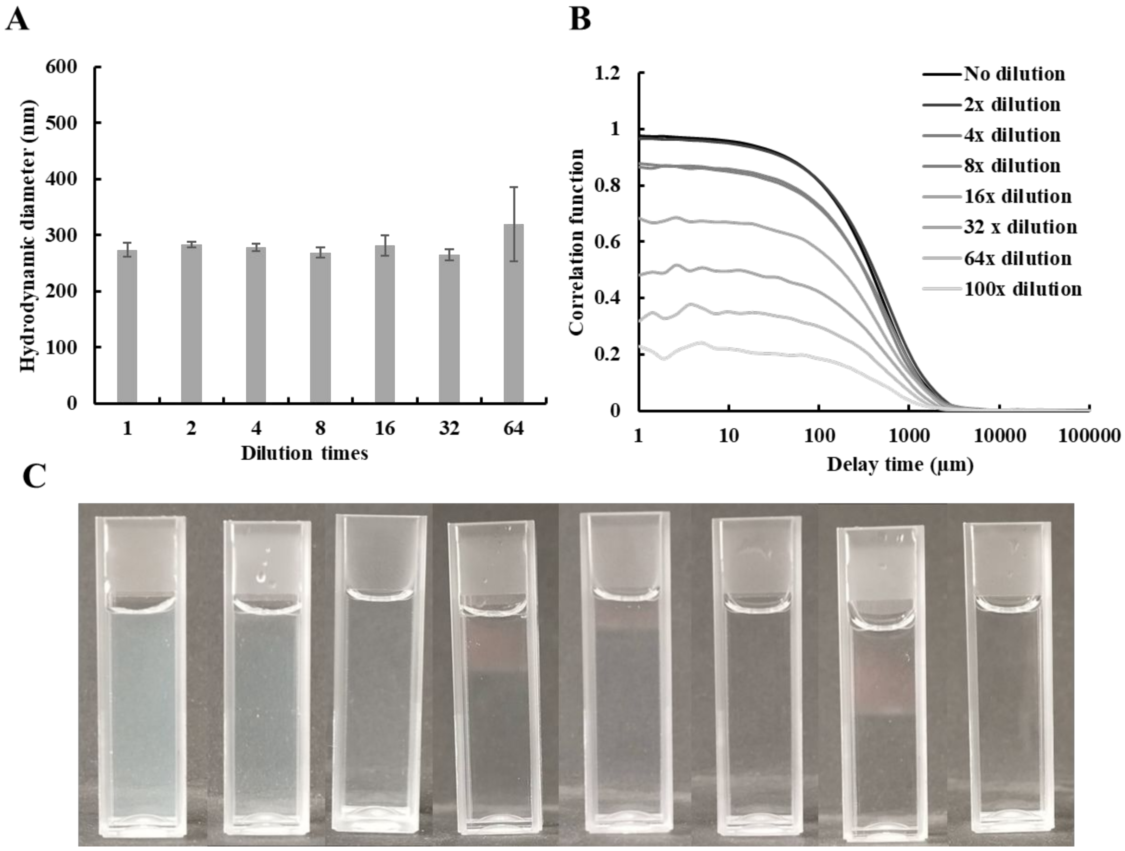

Figure S20. (**A**) The size of the created particles at $m_{Tr}/m_{HyA} = 2$ at different dilutions and (**B**) the correlation functions of the (**C**) measured samples (1, 2, 4, 8, 16, 32, 64, 100 times dilution) *(Photos were taken by László Seres first author)*

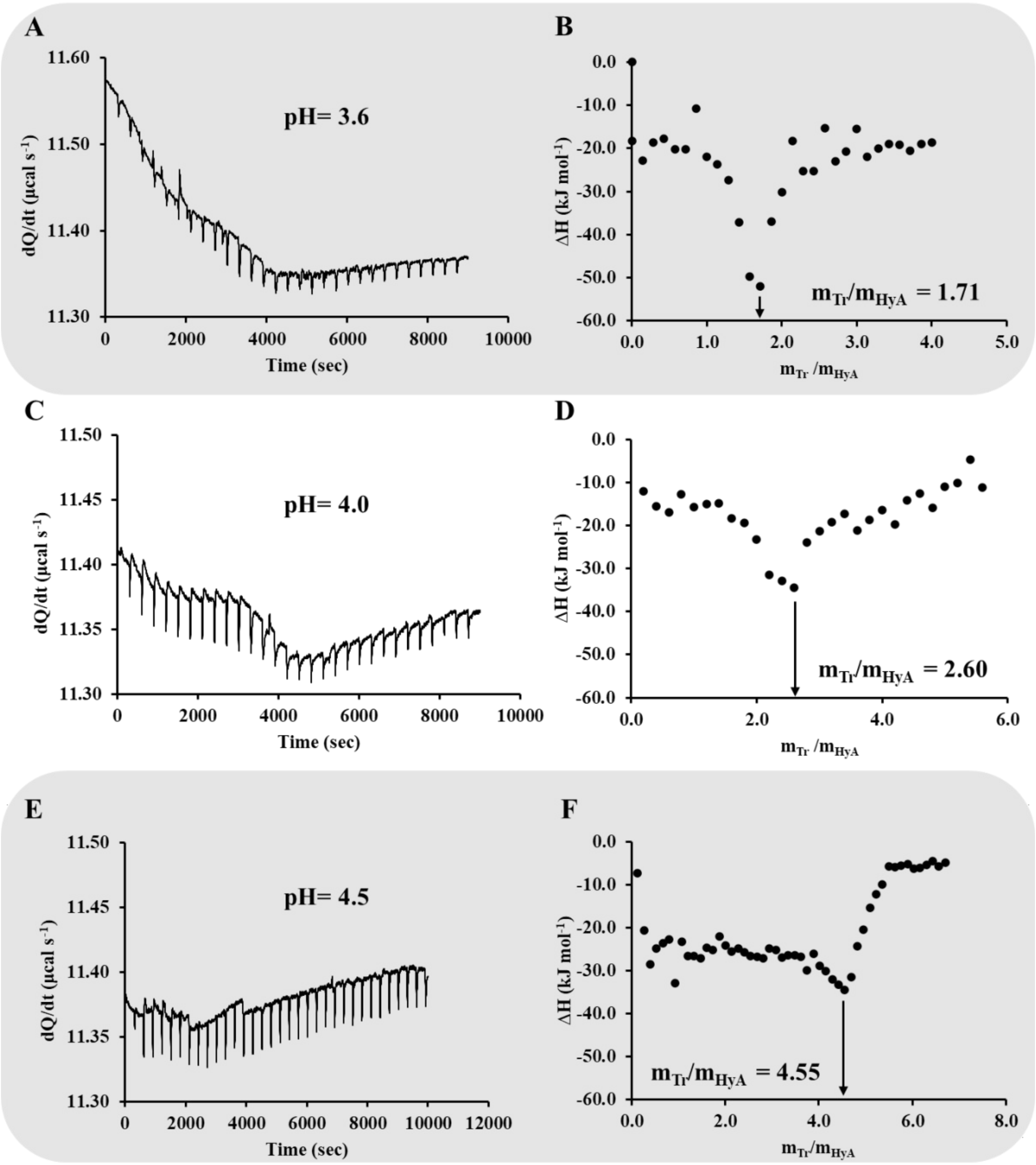

Figure S21. The results of raw calorimetric measurements and the enthalpograms calculated from them at (**A**), (**B**) pH= 3.6 ($c_{Tr\ titrating}$= 1.00 mg/mL), (**C**), (**D**) pH= 4.0 ($c_{Tr\ titrating}$= 1.40 mg/mL) and (**E**), (**F**) pH= 4.5 ($c_{Tr\ titrating}$= 1.875 mg/mL), obtained from the titration of 0.05 mg/mL HyA with Tr

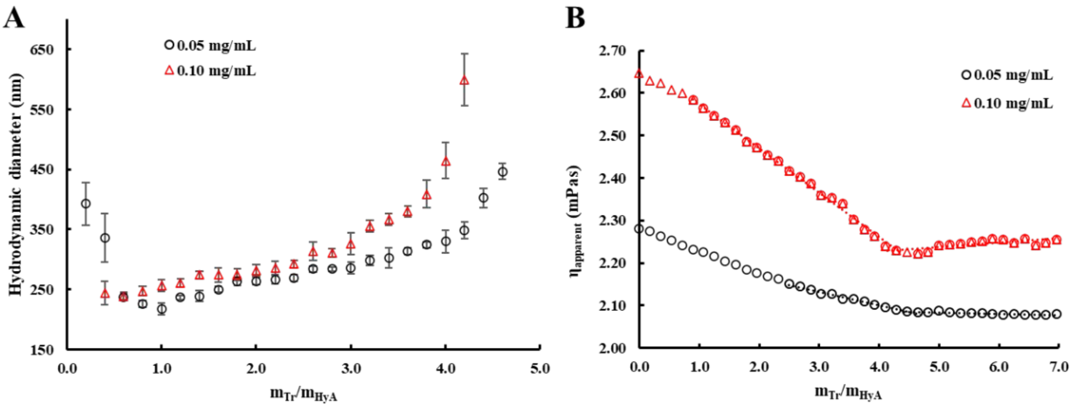

Figure S22. HyA concentration-dependent (**A**) DLS and (**B**) rheological titration curves of the Tr-HyA system ($c_{Tr\ titrating}$= 5 mg/mL, dashed lines represent the linear regression of the viscosimetry data)

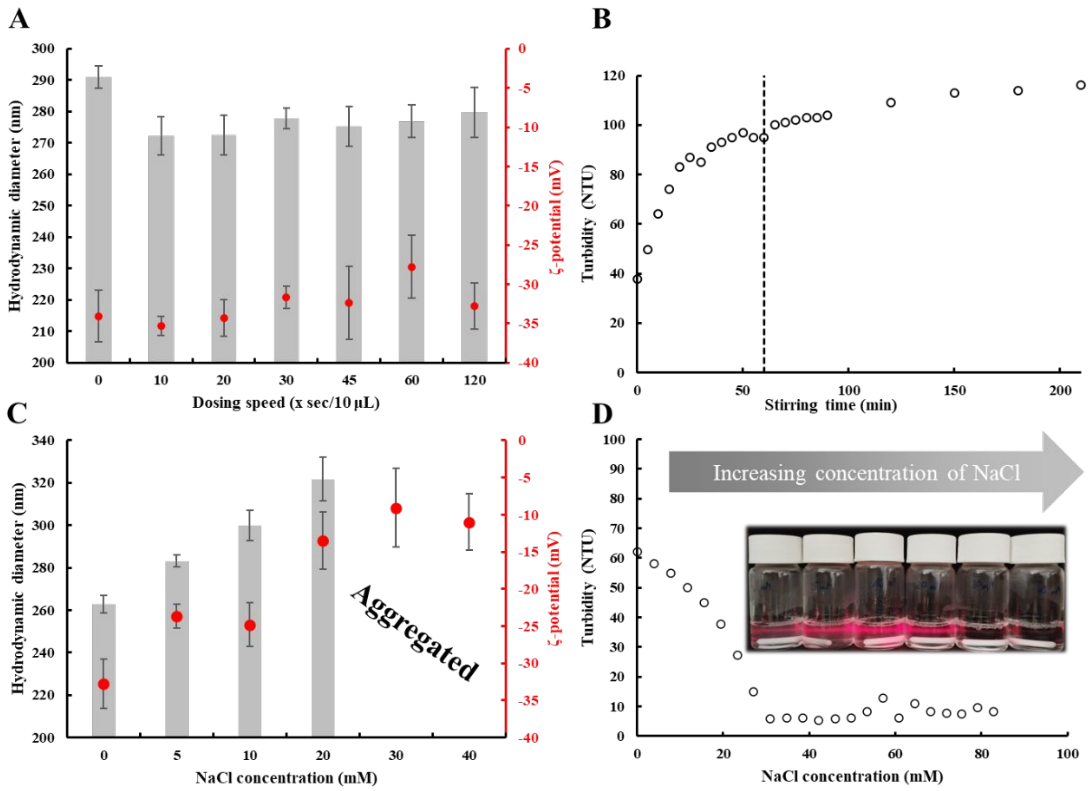

Figure S23. (**A**) The effect of Tr dosing speed on the hydrodynamic size and ζ-potential of the Tr-HyA particles and (**B**) the effect of stirring time on the turbidity of the particle dispersion after preparation. (**C**) The effect of NaCl concentration on the hydrodynamic size and ζ-potential of the Tr-HyA particles and (**D**) the turbidity of the system (c_{HyA} = 0.05 mg/mL, $c_{Tr\ titrating}$ = 5 mg/mL, m_{Tr}/m_{HyA} = 2)

(Photos were taken by László Seres first author)

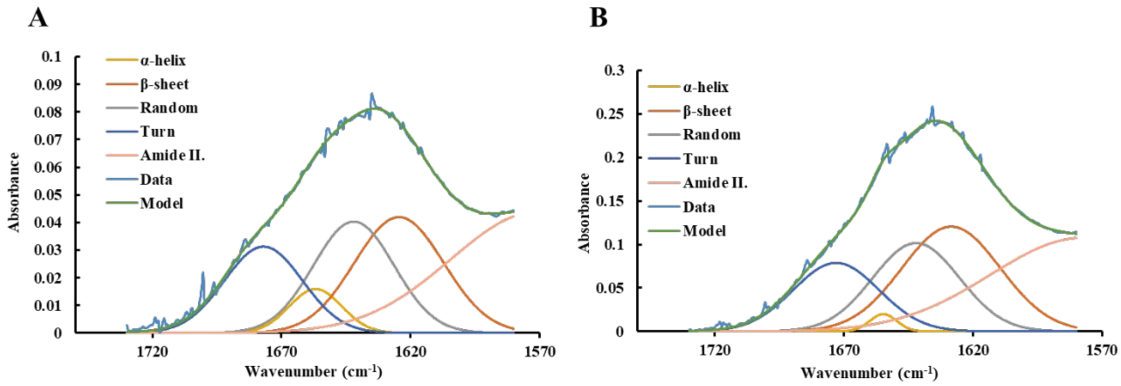

Figure S24. The deconvolution of the amide I. peak for (**A**) the pure Tr protein and (**B**) the Tr-HyA particles

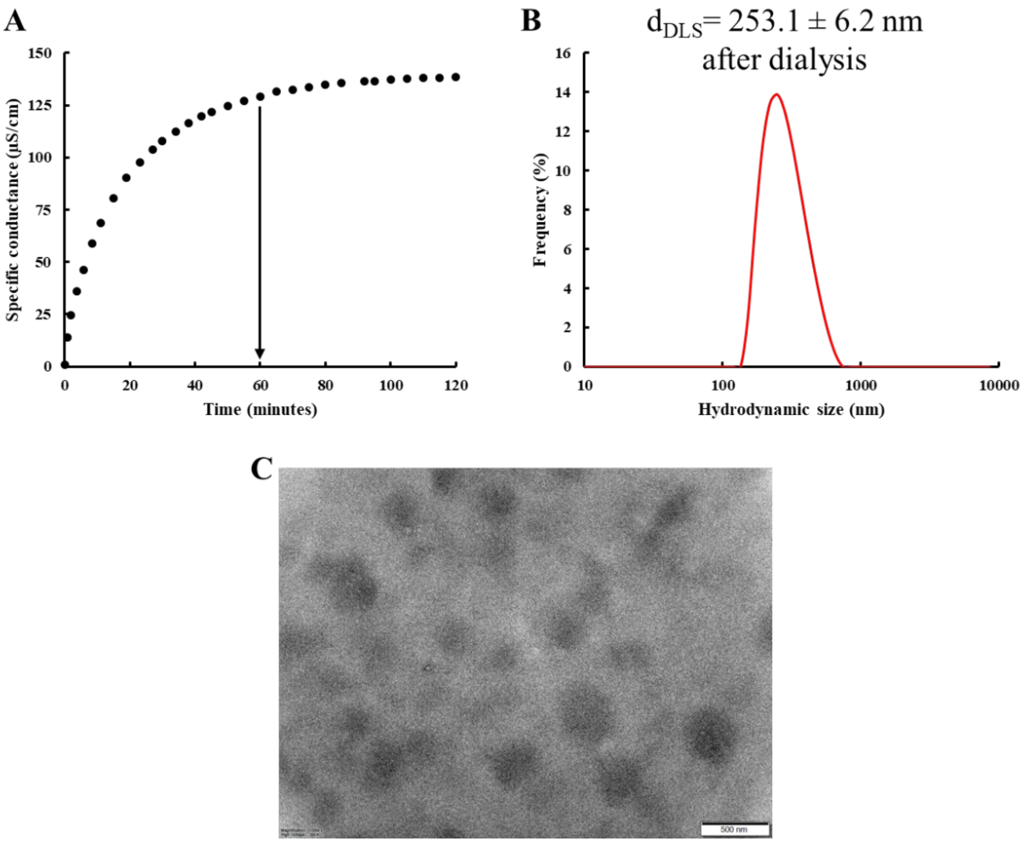

Figure S25. (**A**) The determination of dialysis time, (**B**) the size distribution function of the particles after dialysis and (**C**) a representative TEM image of the particles

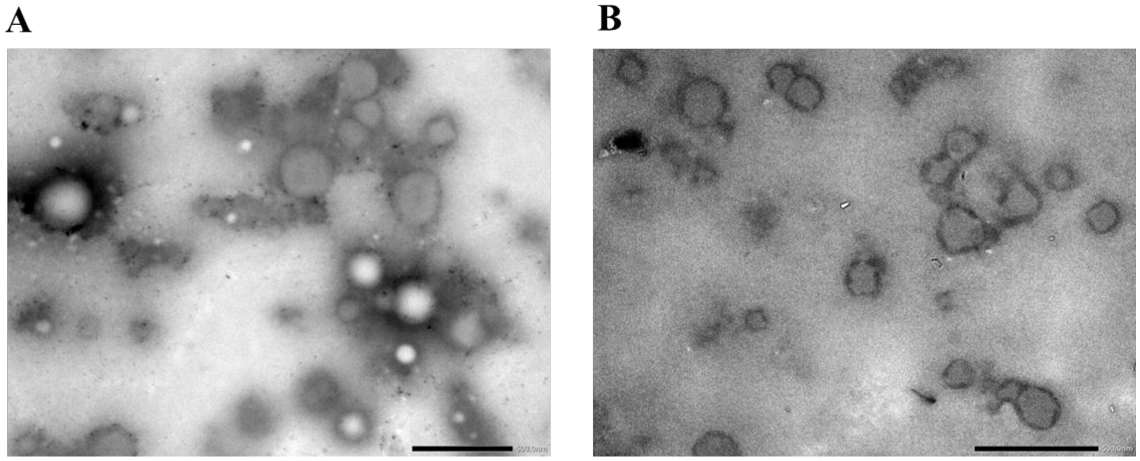

Figure S26. Representative TEM images of the (**A**) Vitamin K1- and (**B**) Vitamin D3-loaded particles ($m_{Tr}/m_{HyA} = 2$, $c_{D3\ and\ K1\ ethanolic\ stock} = 5$ mg/ml)

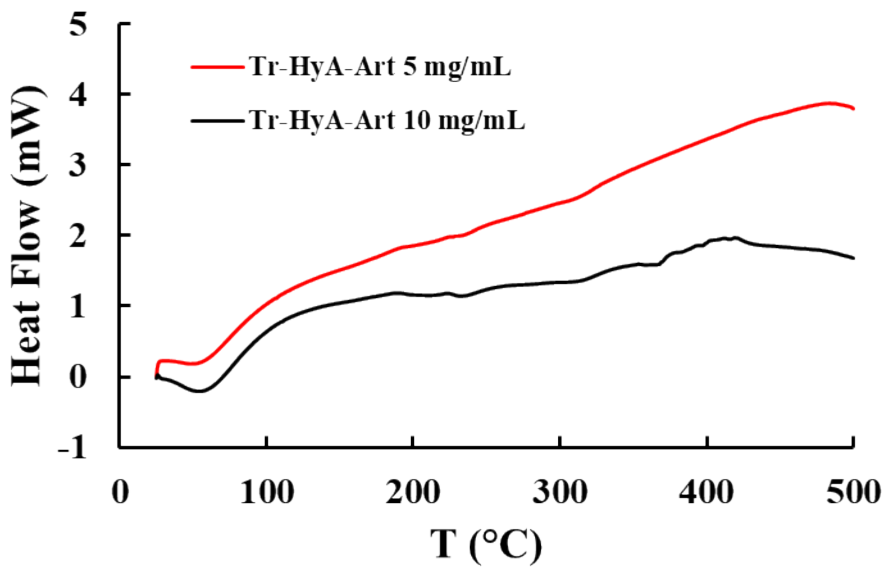

Figure S27. DSC of artesunate samples (m_{Tr}/m_{HyA} = 2, $c_{Art\ ethanolic\ stock}$ = 5 mg/ml and 10 mg/mL)

Table S1. The stability of the unloaded Tr-HyA particles ($m_{Tr}/m_{HyA} = 2$) after 1 hour of dialysis in different media

Medium	pH	d_{DLS} (nm)	PDI
SGF	1.2	7702 ± 1120	3.858 ± 2.43
SIF	6.8	4597 ± 1868	2.113 ± 1.14
PBS	7.4	5331 ± 1667	4.235 ± 3.05
